# PEGylated therapeutics in the clinic

**DOI:** 10.1002/btm2.10600

**Published:** 2023-09-22

**Authors:** Yongsheng Gao, Maithili Joshi, Zongmin Zhao, Samir Mitragotri

**Affiliations:** ^1^ John A. Paulson School of Engineering and Applied Sciences, Harvard University Allston Massachusetts USA; ^2^ Wyss Institute for Biologically Inspired Engineering at Harvard University Boston Massachusetts USA; ^3^ Department of Pharmaceutical Sciences College of Pharmacy, University of Illinois at Chicago Chicago Illinois USA; ^4^ Present address: Department of Bioengineering The University of Texas at Dallas Richardson TX USA

**Keywords:** clinic, clinical translation, clinical trial, drug delivery, FDA, long‐acting drugs, PEGylation, polymer–drug conjugates, protein modification

## Abstract

The covalent attachment of polyethylene glycol (PEG) to therapeutic agents, termed PEGylation, is a well‐established and clinically proven drug delivery approach to improve the pharmacokinetics and pharmacodynamics of drugs. Specifically, PEGylation can improve the parent drug's solubility, extend its circulation time, and reduce its immunogenicity, with minimal undesirable properties. PEGylation technology has been applied to various therapeutic modalities including small molecules, aptamers, peptides, and proteins, leading to over 30 PEGylated drugs currently used in the clinic and many investigational PEGylated agents under clinical trials. Here, we summarize the diverse types of PEGylation strategies, the key advantages of PEGylated therapeutics over their parent drugs, and the broad applications and impacts of PEGylation in clinical settings. A particular focus has been given to the size, topology, and functionalities of PEG molecules utilized in clinically used PEGylated drugs, as well as those under clinical trials. An additional section has been dedicated to analyzing some representative PEGylated drugs that were discontinued at different stages of clinical studies. Finally, we critically discuss the current challenges faced in the development and clinical translation of PEGylated agents.

**Translational Impact Statement**

Over the past 30 years, PEGylation has gained immense popularity and has been proven to be a widely applicable strategy for modifying therapeutics to improve pharmacokinetics and therapeutic efficacy. Recent advancements in PEGylation techniques, coupled with the necessity to overcome challenges such as immunogenicity and polydispersity, present exciting opportunities for the development of novel PEGylated therapeutics. This article provides a comprehensive review of the history and progress of PEGylated therapeutics and emphasizes the significant translational impact PEGylation has achieved. Looking ahead, newer PEGylated therapeutic designs hold promising potential, underscoring the continuous growth and transformative nature of this technique.

## 1 INTRODUCTION

PEGylation has emerged as a widely recognized technology for enhancing the circulation time of various therapeutics, including proteins and small molecules, leading to significant advancements in the development of biologics and drug‐loaded nanoparticles.^1^, ^2^, ^3^ Its impact on the pharmaceutical industry is evident from the large number of PEGylated drugs approved clinically, contributing to a market size of multiple billion US dollars.^4^ Notably, Neulasta® (Amgen), one of the leading PEGylated products, generated sales revenue of $3.2 billion in 2019.^5^ Furthermore, PEGylated lipid nanoparticles (LNPs) were used to formulate the mRNA‐based COVID‐19 vaccines, Comirnaty™ and Spikevax®. In just 2 years, over 8 billion doses of these vaccines have been procured and administered worldwide.^6^, ^7^ These remarkable clinical and commercial success stories of PEGylation have laid a strong foundation for the further expansion of the clinical landscape of PEGylated products, especially in the realm of biologics^8^ and mRNA therapeutics.^9^

In this review, we provide an overview of PEGylation and a comprehensive summary of PEGylated therapeutics that are either approved by the U.S. Food and Drug Administration (FDA) or under active clinical trials. Based on the definition of PEGylation,^10^ we restricted the PEGylated drugs to those therapeutic substances with polyethylene glycol (PEG) molecules attached as “inert” carriers, rather than as part of the active ingredient for the indications. We included PEGylated nanoparticles (NPs), a key frontier of clinically used PEGylated products, although in these cases PEG chains are conjugated to NPs, instead of the bioactive substances. We further discuss the challenges faced in the development of PEGylated drugs from safety, scientific, technological, and translational perspectives as well as some emerging solutions to address these challenges.

## 2 OVERVIEW OF PEGYLATION: HISTORY AND BENEFITS

### 2.1 History

**FIGURE 1:**
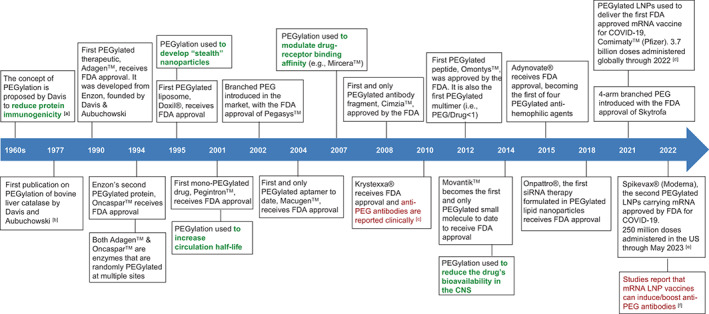
Timeline of the FDA‐approved PEGylated therapeutics and the evolution of therapeutic modalities and PEGylation types. The benefits of PEGylation (in green) and immunogenicity of PEGylated drugs (in red) are also listed. References: Box [a]^11^; Box [b]^12^, ^13^; Box [c]^132^; Box [d]^133^; Box [e]^134^; Box [f].^100^, ^135^, ^136^ CNS, central nervous system; FDA, the U.S. Food and Drug Administration; LNPs, lipid nanoparticles; mRNA, messenger RNA; siRNA, small interfering RNA.

**FIGURE 2:**
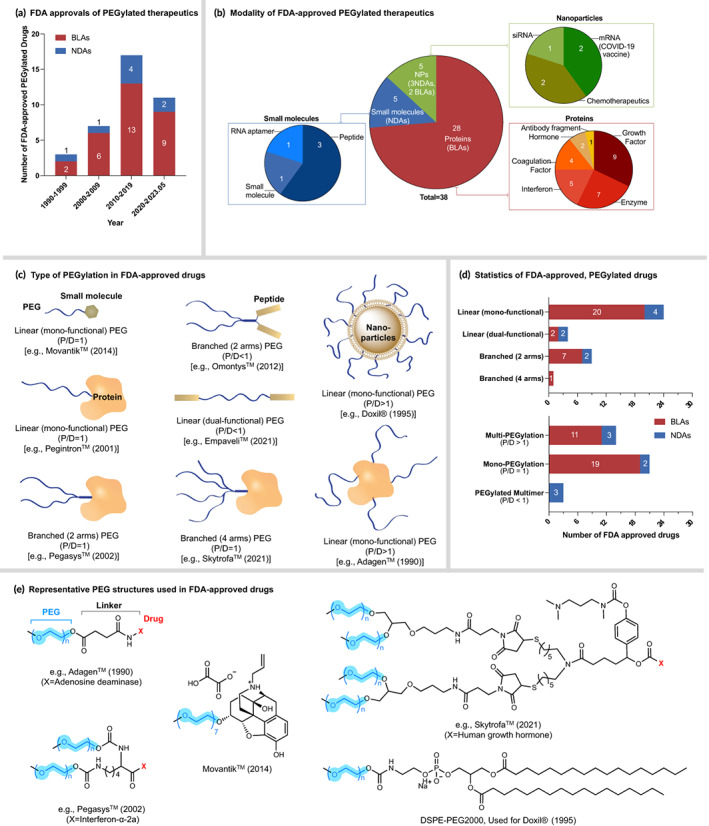
FDA‐approved PEGylated therapeutics. (a) The number of drugs approved per decade. (b) The modality of approved drugs. PEGylated small molecules including peptides and RNA aptamer are approved as new drug applications. PEGylated proteins are approved as biologics license applications. Depending on the modality of the encapsulated drug, PEGylated nanoparticles are approved as either new drug applications or biologics license applications. (c) Types of PEGylation used in FDA‐approved drugs. (d) Statistics of different types of PEGylation. (e) Representative structures of PEG structures used in FDA‐approved drugs. BLAs, biologics license applications; DSPE, 1,2‐Distearoyl‐sn‐glycero‐3‐phosphoethanolamine; FDA, the U.S. Food and Drug Administration; NDAs, new drug applications; NPs, nanoparticles; P/D, the molar ratio of PEG to drug; PEG, polyethylene glycol.

The idea of PEGylation was originally proposed by Frank Davis back in the late 1960s to address the immunogenicity issue of non‐human derived proteins for human use.^11^ PEG was selected because it is the hydrophilic part of a then clinically used block copolymer (Pluronic, consisting of PEG and polypropylene glycol). He hypothesized that the conjugation of PEG to proteins would render them unrecognizable by the immune system as a foreign molecule, thus mitigating immune response against them while enhancing their circulation and activity lifetime. Subsequently, he and Abraham Aubuchowski published the first research article on a PEGylated enzyme, bovine liver catalase, showing that this modified enzyme did indeed have lower immunogenicity and a longer circulation half‐time.^12^, ^13^ They continued their work on PEGylation and in 1981 founded the first PEGylation company, Enzon. The first two PEGylated products to receive FDA approval were Adagen™ (1990) and Oncaspar™ (1994), both marketed by Enzon and using PEGylation to extend the half‐life of the active enzymes. Following these two early successes, there was a rapid rise in the number of PEGylated products that entered the market, marking the introduction of different therapeutic modalities, different PEG architectures as well as different PEG/drug ratios. The timeline and evolution of PEGylation has been depicted in Figure 1 and the FDA‐approval trends of PEGylated drugs observed over the years are shown in Figure 2a. In just over three decades, the field has seen the approvals of the first PEGylated protein (Adagen™, 1990), the first PEGylated liposome (Doxil®, 1995), the first PEGylated aptamer (Macugen™, 2004), the first PEGylated antibody fragment (Cimzia™, 2008), the first PEGylated peptide (Omontys™, 2012), the first PEGylated small molecule (Movantik™, 2014), the first PEGylated siRNA therapeutic (Onpattro™, 2018) and notably, the two recent PEGylated LNP‐mRNA‐based vaccines (Comirnaty™, 2021; Spikevax®, 2022).^14^ The PEGylation technology is rapidly evolving and new PEGylated therapeutics are continuously emerging.^15^, ^16^, ^17^ It should be noted that the global rollout of these PEGylated LNP‐mRNA‐based vaccines has had a profound impact on the clinical landscape of PEGylation as a drug delivery technology, since it demonstrated the possibility to manufacture, distribute and administer billions of doses of PEGylated nanoparticles to a massive population.

### 2.2 Physicochemical properties and benefits

PEG, consisting of repeated ethylene oxide units, is highly hydrophilic and flexible, due to the strong interactions between water molecules and ether oxygens distributed along the polymer backbone. The high hydration and flexibility of PEG are the molecular foundation for the clinical benefits observed in the PEGylated products, in addition to other properties such as non‐toxicity and biocompatibility.^12^, ^13^

PEGylation was originally developed to shield proteins from the immune system, because PEG and its bound water can form a flexible hydrophilic shell to cover the antigenic determinants. As such, PEGylation reduced the generation of neutralizing antibodies against the enzymes and other adverse immune responses.^18^ This laid the foundation for the development and subsequent approval of Adagen™ and Oncaspar™. However, since then, the benefits of PEGylation have been substantially expanded.

Pegintron™ and Pegasys™, two approved PEGylated products, relied on PEGylation to extend the circulating half‐life of the parent proteins, interferons, for the treatment of Hepatitis C. The modification of interferon alfa‐2a with a 40 kDa PEG in Pegasys™ resulted in sustained absorption and reduced renal clearance, subsequently leading to superior therapeutic efficacy to the free drug, interferon alfa‐2a.^19^, ^20^ An increase in half‐life of the free drug due to PEGylation is a benefit that can be attributed to the increase in the hydrodynamic size and subsequent decrease in renal filtration due to PEG conjugation. This is a property that several marketed technologies have exploited, including Somavert™ (half‐life increase from 20 min to 72 h),^21^ Neulasta® (from 3.5 to 15–80 h)^22^, ^23^ and Krystexxa® (from <24 h to 2 weeks).^24^, ^25^

PEGylation has also been used to modulate the drug's activity at the receptor level. For instance, Mircera™, a PEGylated epoetin‐beta product, has a slower association and faster dissociation rate from its corresponding receptor as well as an increased half‐life, all of which contribute to its less frequent dosing regimen as compared to the parent drug.^26^ Adynovate® is another example in which PEG plays a role in changing the mechanism of action of the drug. Following PEGylation, FVIII was found to retain all the physiological functions of free FVIII, with a reduced binding to the low‐density lipoprotein receptor‐related protein clearance receptor, resulting in slower clearance and prolonged circulation.^27^

In the case of the small molecular drug, Movantik™ (naloxegol, PEGylated naloxone), PEGylation was used to reduce its bioavailability in the central nervous system (CNS). The presence of PEG in naloxegol reduces its passive permeability when compared to free naloxone, thus resulting in negligible penetration of the CNS via the blood–brain barrier. PEGylation also makes naloxegol a substrate for the P‐glycoprotein receptor, the effect of which is an increased efflux of naloxegol across the blood–brain barrier. This reduces opioid‐induced constipation in the gastrointestinal tract, while still retaining its central analgesic effect.^28^

The presence of PEG on the surface of the liposomal carriers in the marketed product Doxil® has been shown to reduce their uptake by the mononuclear phagocyte system (MPS), thus imparting “stealth” properties to the liposome and resulting in prolonged circulation time.^29^ In the case of LNPs such as Comirnaty™ and Spikevax®, PEGylation is used to enhance stability, decrease aggregation and protect the LNPs from uptake by the MPS. It also provides a “stealth effect” that decreases protein adsorption on the surface of nanoparticles.^30^, ^31^, ^32^

To summarize, PEGylation has been shown to increase the molecular mass and hydrodynamic sizes of small molecules and peptides, play a role in receptor binding, decrease aggregation of nanoparticles, and shield proteins and peptides from proteolytic enzymes and neutralizing antibodies, thus improving pharmacokinetics of the drug. This, in turn, has led to more effective and safer therapeutics with increased patient compliance.

## 3 FDA‐APPROVED PEGYLATED THERAPEUTICS

To date, a total of 38 PEGylated therapeutics have been approved by the FDA as either new drug applications (NDAs) or biologics license applications (BLAs) as listed in Table 1. The number of approved PEGylated therapeutics is expected to grow rapidly, given the significant pre‐clinical research activities in the field of PEGylation and the exponential growth of FDA‐approval trends of PEGylated drugs observed over the years (Figure 2a). In this section, we summarize the modality of PEGylated therapeutics approved by the FDA, their approved and investigational indications and types of PEGylation (e.g., PEG topology and size) with the aim to provide a clinical landscape of current PEGylated drugs and a guideline for the further design of PEGylated molecules.

**TABLE 1:** FDA approved PEGylated therapeutics.

Trade name [generic name]	Approval holder	Parent drug	Drug size (kDa)	PEG size (kDa)	Mode of action of PEG	PEG topology	P/D	Indication	Dosing route	Identifier (year)
Proteins										
Elfabrio® [Pegunigalsidase alfa‐iwx]	Chiesi Farmaceutici S.p.A	Human α‐galactosidase‐A	98 (estimated, dimer)	2.3	Enzyme stability enhancement	Linear (dual‐functional)	9	Fabry disease	IV	BLA 761161 (2023)
Fylnetra™ [Pegfilgrastim‐pbbk]	Amneal Pharmaceuticals LLC	G‐CSF	19	20	Increase in circulating half‐life	Linear	1	Infection during chemotherapy	SC	BLA 761084 (2022)
Stimufend® [Pegfilgrastim‐fpgk]	Fresenius Kabi	G‐CSF	19	20	Increase in circulating half‐life	Linear	1	Infection during chemotherapy	SC	BLA 761173 (2022)
Rolvedon™ [Eflapegrastim‐xnst]	Spectrum Pharmaceuticals	G‐CSF	18.6	3.4	Linkage between G‐CSF and an Fc fragment of immunoglobulin G4	Linear (dual‐functional)	1	Infection during chemotherapy	SC	BLA 761148 (2022)
Skytrofa™ [Lonapegsomatropin‐tcgd]	Ascendis	Human growth hormone (Somatropin)	22	40	Increase in circulating half‐life	Branched (4 arms)	1	Growth hormone deficiency	SC	BLA 761177 (2021)
Besremi™ [Ropeginterferon alfa‐2b‐njft]	PharmaEssentia Corp	Interferon‐α‐2b	20	40	Increase in circulating half‐life	Branched (2 arms)	1	Polycythemia vera	SC	BLA 761166 (2021)
Nyvepria™ [Pegfilgrastim‐apgf]	Pfizer, Inc.	G‐CSF	19	20	Increase in circulating half‐life	Linear	1	Infection during chemotherapy	SC	BLA 761111 (2020)
Esperoct® [Turoctocog alfa pegol]	Novo Nordisk	Coagulation Factor VIII	166	40	Increase in circulating half‐life	Branched (2 arms)	1	Hemophilia A	IV	BLA 125671 (2019)
Ziextenzo™ [Pegfilgrastim‐bmez]	Sandoz	G‐CSF	19	20	Increase in circulating half‐life	Linear	1	Infection during chemotherapy	SC	BLA 761045 (2019)
Jivi™ [Damoctocog alfa pegol]	Bayer Healthcare	Coagulation Factor VIII (B‐domain deleted)	169	60	Increase in circulating half‐life	Branched (2 arms)	1	Hemophilia A	IV	BLA 125661 (2018)
Palynziq™ [Pegvaliase‐pqpz]	BioMarin Pharmaceutical	Phenylalanine ammonia‐lyase	248 (tetramer)	20	Reduction in immune recognition	Linear	36	Phenylketonuria	SC	BLA 761079 (2018)
Revcovi™ [Elapegademase‐lvlr]	Leadiant Bioscience	Adenosine deaminase	33	5.6	Reduction in immune recognition	Linear	13	ADA‐SCID	IM	BLA 761092 (2018)
Asparlas™ [Calaspargase pegol‐mknl]	Servier Pharma	L‐asparaginase	138 (tetramer)	5	Increase in circulating half‐life and reduction in immune recognition	Linear	31–39	Acute lymphoblastic leukemia	IV	BLA 761102 (2018)
Fulphila™ [Pegfilgrastim‐jmdb]	Mylan GmbH	G‐CSF	19	20	Increase in circulating half‐life	Linear	1	Infection during chemotherapy	SC	BLA 761075 (2018)
Udenyca™ [Pegfilgrastim‐cbqv]	Coherus Biosciences	G‐CSF	19	20	Increase in circulating half‐life	Linear	1	Infection during chemotherapy	SC	BLA 761039 (2018)
Rebinyn® [Nonacog beta pegol]	Novo Nordisk	Coagulation Factor lX	56	40	Increase in circulating half‐life	Branched (2 arms)	1	Hemophilia B	IV	BLA 125611 (2017)
Adynovate® [Rurioctocog alfa pegol]	Baxalta	Coagulation Factor VIII (ADVATE)	280	20	Increase in circulating half‐life	Branched (2 arms)	2 (estimated)	Hemophilia A	IV	BLA 125566 (2015)
Plegridy™ [Peginterferon beta‐1a]	Biogen	Interferon β‐1a	20	20	Increase in circulating half‐life	Linear	1	Multiple sclerosis	SC	BLA 125499 (2014)
Sylatron™ [Peginterferon alfa‐2b]	Merck	Interferon‐α‐2b	19.3	12	Increase in circulating half‐life	Linear	1	Melanoma	SC	BLA 103949 (2011)
Krystexxa® [Pegloticase]	Horizon Pharma	Urate oxidase	137 (tetramer)	10	Reduction in immunogenicity	Linear	40	Chronic gout	IV	BLA 125293 (2010)
Cimzia™ [Certolizumab pegol]	UCB, Inc.	anti‐TNFα Fab'	51	40	Increase in circulating half‐life	Branched (2 arms)	1	Crohn's Disease, Rheumatoid arthritis, Psoriatic arthritis, Ankylosing spondylitis	SC	BLA 125160 (2008)
Mircera™ [Methoxy polyethylene glycol‐epoetin beta]	Roche	Erythropoietin	30	30	Increase in circulating half‐life	Linear	1	Anemia associated with chronic kidney disease	SC	BLA 125164 (2007)
Somavert™ [Pegvisomant]	Pfizer	Human growth hormone	22	5	Increase in circulating half‐life	Linear	4–6	Acromegaly	SC	BLA 021106 (2003)
Neulasta® [Pegfilgrastim]	Amgen	G‐CSF	19	20	Increase in circulating half‐life	Linear	1	Infection during chemotherapy	SC	BLA 125031 (2002)
Pegasys™ [Peginterferon alfa‐2a]	Roche	Interferon‐α‐2a	20	40	Increase in circulating half‐life	Branched (2 arms)	1	Chronic hepatitis C, Chronic hepatitis B, Cirrhosis and compensated liver disease, CHC/HIV coinfection	SC	BLA 103964 (2002)
Pegintron™ [Peginterferon alfa‐2b]	Schering	Interferon‐α‐2b	19.3	12	Increase in circulating half‐life	Linear	1	Chronic hepatitis C	SC	BLA 103949 (2001)
Oncaspar™ [Pegaspargase]	Enzon	L‐asparaginase	138 (tetramer)	5	Increase in circulating half‐life and reduction in immune recognition	Linear	69–82	Acute lymphoblastic leukemia	IM, IV	BLA 103411 (1994)
Adagen™ [Pegademase bovine]	Enzon	Adenosine deaminase	33	5	Reduction in immune recognition	Linear	11–17	ADA‐SCID	IM	BLA 019818 (1990)/Discontinued
Small molecules										
Syfovre™ [Pegcetacoplan]	Apellis Pharmaceuticals, Inc.	Complement C3 inhibitor peptide	1.75	40	Increase in circulating half‐life	Linear (dual‐functional)	0.5	Geographic atrophy secondary to AMD	IVT	NDA 217171 (2023)
Empaveli™ [Pegcetacoplan]	Apellis Pharmaceuticals, Inc.	Complement inhibitor peptide	1.75	40	Increase in circulating half‐life	Linear (dual‐functional)	0.5	Paroxysmal nocturnal hemoglobinuria	SC	NDA 215014 (2021)
Movantik® [Naloxegol]	AstraZeneca	Naloxone	0.418	0.323	Reduced permeability into CNS	Linear	1	Opioid‐induced constipation	PO	NDA 204760 (2014)
Omontys™ [Peginesatide]	Takeda	Erythropoietin mimetic peptide	2.45	40	Increase in circulating half‐life	Branched (2 arms)	0.5	Anemia due to chronic kidney disease	IV, SC	NDA 202799 (2012)/Discontinued
Macugen™ [Pegaptanib sodium]	Pfizer	RNA aptamer	10 (estimated)	40	Increase in intravitreal residence time	Branched (2 arms)	1	Neovascular (wet) age‐related macular degeneration	IVT	NDA 021756 (2004)/Discontinued
Nanoparticles										
Spikevax® [COVID‐19 Vaccine, mRNA]	Moderna	mRNA in LNPs	–	2	Reduction in protein adsorption and phagocytic clearance	Linear (on NPs)	Multiple	COVID‐19	IM	BLA 125752 (2022)
Comirnaty™ [COVID‐19 Vaccine, mRNA]	BioNTech/Pfizer	mRNA in LNPs	–	2	Reduction in protein adsorption and phagocytic clearance	Linear (on NPs)	Multiple	COVID‐19	IM	BLA 125742 (2021)
Onpattro® [Patisiran]	Alnylam Pharmaceuticals	siRNA in LNPs	–	2	Reduction in protein adsorption and phagocytic clearance	Linear (on NPs)	Multiple	Polyneuropathy of hereditary transthyretin‐mediated amyloidosis	IV	NDA 210922 (2018)
Onivyde™ [Irinotecan liposome]	Merrimack Pharmaceuticals	Irinotecan in liposome	–	2	Reduction in protein adsorption and phagocytic clearance	Linear (on NPs)	Multiple	Metastatic adenocarcinoma of the pancreas post gemcitabine treatment	IV	NDA 207793 (2015)
Doxil® [Doxorubicin HCl liposome]	Schering	Doxorubicin in liposome	–	2	Reduction in protein adsorption and phagocytic clearance	Linear (on NPs)	Multiple	Ovarian cancer, Multiple myeloma, AIDS‐related Kaposi's Sarcoma	IV	NDA 050718 (1995)

Abbreviations: BLAs, biologics license applications; DSPE, 1,2‐Distearoyl‐sn‐glycero‐3‐phosphoethanolamine; FDA, the U.S. Food and Drug Administration; G‐CSF, granulocyte colony‐stimulating factor; IV, intravenous injection; IVT, intravitreal injection; IM, intramuscular injection; NDAs, new drug applications; NPs, nanoparticles; P/D, the molar ratio of PEG to drug; PEG, polyethylene glycol; PO, per os or by mouth; SC, subcutaneous.

### 3.1 Modality of PEGylated drugs

Overall, the majority of PEGylated therapeutics^33^ are based on therapeutic proteins, with 28 PEGylated proteins received FDA approvals (Figure 2b), accounting for 74% of approved PEGylated drugs and 93% of the PEGylated BLAs. This is reasonable as the key motivation of PEGylation is to increase the half‐life of proteins. One major type of protein that has succeeded in PEGylating is the growth factor, including granulocyte colony‐stimulating factor (G‐CSF) (e.g., Rolvedon™, Neulasta®, Nyvepria™, Ziextenzo™, Udenyca™, Stimufend®, Fulphila™, and Fylnetra™) and erythropoietin (EPO) (e.g., Mircera™). Neulasta® (pegfilgrastim), developed as a long‐acting form of filgrastim, is the first FDA‐approved PEGylated G‐CSF. Since its initial launch in 2002, there have been six PEGylated G‐CSF drugs approved as biosimilars to Neulasta®. Udenyca™ (pegfilgrastim‐cbqv), which is the historic second biosimilar approved by the FDA, was further approved as an interchangeable biosimilar to Neulasta® in 2022.^33^ Rolvedon™ (eflapegrastim‐xnst) is another novel, long‐acting G‐CSF, approved in 2022. Unlike other PEGylated proteins, Rolvedon™ was developed using a dual‐functional PEG as a linear linker to connect the recombinant human G‐CSF analog with an Fc fragment of human immunoglobulin G4, which was selected to increase its half‐life and uptake to the bone marrow.^34^ Therapeutic enzymes are another popular type of protein seeking PEGylation, leading to the FDA approvals of seven PEGylated enzymes so far. Enzymes used in these therapeutics include adenosine deaminase (Adagen™ and Revcovi™), L‐asparaginase (Oncaspar™ and Asparlas™), α‐Galactosidase A (Elfabrio®), phenylalanine ammonia‐lyase (Palynziq™) and uricase (Krystexxa®). Among them, Adagen™ used an enzyme derived from bovine intestine and was discontinued due to the shortage of the therapeutic enzyme. PEGylated interferons and coagulation factors have also been developed as immunostimulant and antihemorrhagic, respectively. Although antibody‐based therapeutics have received significant attention recently, only one PEGylated antibody fragment (Cimzia™) has received FDA approval, presumably due to the moderate necessity of extending the circulation time of such proteins.

In terms of small molecular drugs, five PEGylated drugs (including peptides and RNA aptamer) have been approved as NDAs, as shown in Figure 2b. Three of them are peptide‐based therapeutics, including pegcetacoplan (Empaveli™ and Syfovre™) as an immunosuppressant and peginesatide (Omontys™) as an erythropoiesis stimulator. Another notable PEGylated small molecular drug is Macugen™ (pegaptanib sodium, Pfizer), which is a PEGylated aptamer, functioning as a selective vascular endothelial growth factor antagonist. It is the first aptamer‐based therapeutic approved for human use.^35^ Besides these novel therapeutics, Movantik™ (naloxegol) is the only PEGylated drug consisting of a traditionally considered small molecule: naloxone (Figure 2e). It was approved by the FDA in 2014 as an opioid antagonist. The short PEG moiety (7 repeating units) is introduced to reduce the passive permeability of naloxone and thereby achieving negligible penetration into the CNS.

Instead of directly conjugating to therapeutic molecules, attaching PEG molecules to NP‐based drug delivery systems has also been demonstrated as an effective and feasible strategy to extend their half‐life and provide stealth effect (Figure 2b). Since the first approval of Doxil® (doxorubicin HCl liposome) by the FDA in 1995, another four PEGylated NPs have been translated into the clinic, including the two recent mRNA‐based COVID‐19 vaccines: Comirnaty™ and Spikevax®. Doxil® and Onivyde™ are two PEGylated liposome formulations, developed to formulate the chemotherapeutics doxorubicin and irinotecan, respectively. On the other hand, Comirnaty™, Spikevax® and Onpattro® are LNP‐based formulations for RNA therapeutics. For Onpattro®, the active ingredient is small interfering RNA, which can cause degradation of transthyretin mRNA via RNA interference, thereby lowering the serum transthyretin protein levels. Onpattro®, Doxil® and Onivyde™ were approved as NDAs, while Comirnaty™ and Spikevax® were approved as BLAs.

### 3.2 Types of PEGylation

Over the years, a diverse set of PEG molecules and conjugation strategies have been developed for PEGylation and many of them have entered the clinic. In this subsection, we summarize different types of PEGylation used in FDA‐approved drugs by summarizing three structural parameters: PEG topology (i.e., linear or branched), PEG to drug molar ratio (i.e., multi‐, mono‐, or PEGylated multimer) and sizes of PEG and therapeutic agent (Figure 2c,d). These three parameters were chosen because: (i) it is known that both the size and topology determine the hydrodynamic volumes of PEG, which further regulate the circulation time and excretion routes of individual PEG molecules; (ii) the circulation half‐life of PEGylated molecules will thereby be influenced by the hydrodynamic volumes of the individual PEG and the number of PEG chains in each agent.^8^, ^10^ Some representative PEG structures used in FDA‐approved drugs are shown in Figure 2e.

Attaching multiple strands of linear, monofunctional PEG to one therapeutic molecule represents the first generation of PEGylation technology, which was used in developing the first two FDA‐approved drugs: Adagen™ and Oncaspar™. So far, a total of seven drugs have been developed using this strategy, and all of them are protein‐based biologics. The most recent ones are Palynziq™ (pegvaliase‐pqpz), Revcovi™ (elapegademase‐lvlr), and Asparlas™ (calaspargase pegol‐mknl), approved by the FDA in 2018. The five PEGylated NP‐based therapeutics can also fall into this category since each NP is multi‐PEGylated with linear monofunctional PEG moieties. Another multi‐PEGylation strategy is based on linear, bifunctional PEG to covalently crosslink two protein molecules, leading to the recently approved biologic Elfabrio® (pegunigalsidase alfa‐iwx) in 2023. It was developed through reactions between N‐hydroxysuccinimide on the two ends of bifunctional PEG and the amines of lysine on proteins, and about nine PEGs were attached to the homodimeric protein. It was assumed that only a part of the nine bifunctional PEGs are reacted on both sides for crosslinking and the rest of PEGs are attached to the protein as dangling chains.^36^ Compared to the monofunctional PEG‐based conjugates, bifunctional PEG crosslinked proteins showed a higher enzyme reactivity and stability in vitro.^36^ Adynovate® (rurioctocog alfa pegol) is the only nonlinear PEG‐based, multi‐PEGylated drug, that utilized a 2‐arm branched PEG with the size of 10 kDa on each arm.^37^ Based on its FDA‐approved label, one or more PEGs are conjugated to the parent protein, human coagulation factor VIII. Overall, the sizes of PEGs used in this strategy are relatively small (<10 kDa), with one exception, Palynziq™ (20 kDa PEGs). Such multi‐PEGylated proteins are generally heterogeneous in structure with a varied amount of PEG attached to proteins at non‐specific sites, representing one of the major technological limitations of such PEGylation for clinical translation.

The approval of Pegintron™ (peginterferon alfa‐2b, 2001) and Neulasta® (pegfilgrastim, 2002) marked the initiation of site‐specific mono‐PEGylation. This is an exciting phase of the PEGylation technology that has received considerable attention in recent years. This technology in principle can generate homogenous mono‐PEGylated products, which is generally preferred for large‐scale manufacturing, quality control and regulatory approval. The following two decades witnessed the approval of another 10 mono‐PEGylated drugs using linear PEGs (Table 1), including the first and only small‐molecule‐based PEGylated drug, Movantik™ (naloxegol, 2014). PEG moieties in these drugs, except for Movantik™, are typically larger (>12 kDa) than those for multi‐PEGylation. Other than linear PEGs, the first branched PEG‐based mono‐PEGylated drug, Pegasys™ (peginterferon alfa‐2a), was approved in 2002. Such branched PEGs, including a 4‐arm branched one (used for Skytrofa™ (lonapegsomatropin‐tcgd)), have been used in a total of eight approved mono‐PEGylated drugs. Among them, only Jivi™ (damoctocog alfa pegol) consists of a 60 kDa PEG, while others are all 40 kDa‐sized PEGs.

Unlike multi‐ or mono‐PEGylation mentioned above, attaching multiple drugs onto one PEG molecule, resulting in a PEG/drug ratio of <1, is another type of PEGylation. As listed in Table 1, three of such PEGylated peptide‐based therapeutics have been approved, and all of them consist of two peptides connected to one PEG moiety. We termed them as PEGylated multimers given that multiple drugs are connected to one PEG molecule. Among these PEGylated multimers, Omontys™ (peginesatide) has a 2‐arm branched PEG with two peptides attached at each side of the PEG, while pegcetacoplan (Syfovre™ and Empaveli™) contains a linear PEG with two peptides conjugated to both sides. This strategy is distinct and useful in increasing the content of active substances in the product, especially for small molecular entities.

Most of the PEG molecules that have the therapeutic conjugated to one end, are unmodified at the other end and have an inert methoxy group at the non‐conjugated terminal. This is the case for all PEGylated nanoparticles and majority of the PEGylated small molecules and proteins. When the PEG is used as a linker between the therapeutic as in the case of Elfabrio™, both the end groups of the PEG originally functionalized with a bis‐NHS ester are involved in conjugation.

**FIGURE 3:**
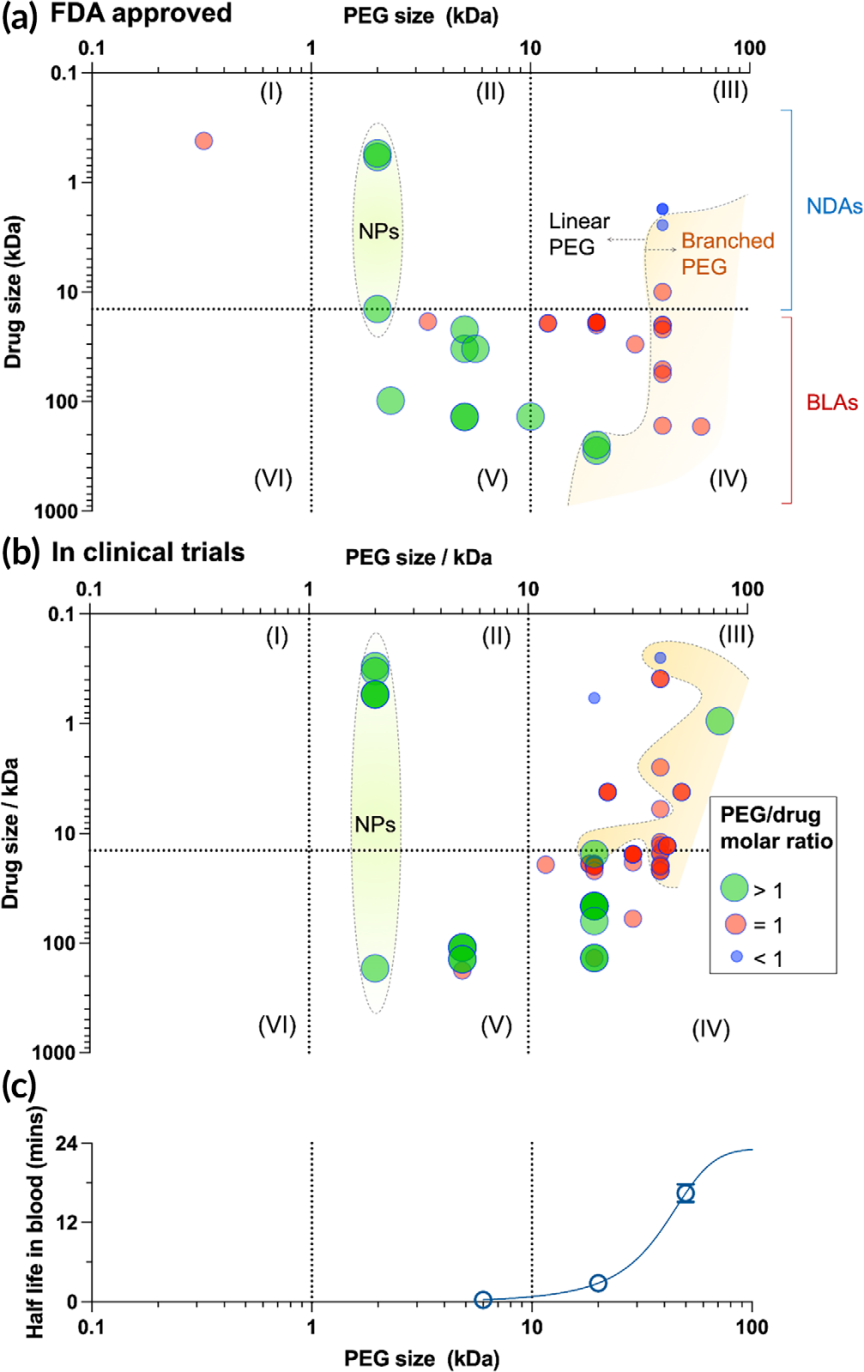
Groups of PEGylated agents based on sizes of PEG and parent drugs. PEG sizes are segmented at 1 and 10 kDa (vertical dash line), while the horizontal dash line indicates the largest molecular weight of parent drugs (Patisiran, 14.3 kDa) approved as NDAs. (a) FDA‐approved PEGylated drugs span into five groups (I–V). (b) Investigational PEGylated agents under active clinical trials distributed in Group II–V. (c) Half‐lives of native PEG molecules in blood after intravenous administration depends on their sizes. Data are based on a murine study from Reference 38. (PEG of 170 kDa is out of the molecular weight range and thus is not shown here). The solid line is fitted using a Sigmoidal model (4PL).

In terms of PEG sizes used for these PEGylation strategies, we cross‐compared the sizes of PEG and the corresponding active substance of these FDA‐approved therapeutics and further categorized them into six groups, as shown in Figure 3a. The two mRNA‐based COVID‐19 vaccines (Spikevax® and Comirnaty™) were not shown, because the molecular weights of mRNAs used were not found. Movantik™ is the only PEGylated drug carrying a PEG moiety with size of less than 1 kDa (Type I). While rare, this is an interesting type of PEGylation that can potentially generate drugs with specific, predefined chemical structures and identical sizes, given the commercial access of monodisperse PEGs in this size range. However, further efforts are needed to integrate such short PEGs with therapeutic agents in a way that can achieve therapeutic benefits (e.g., improving pharmacokinetics). PEG with moderate molecular weight (1–10 kDa) are mainly used for multi‐PEGylation (green circles in Figure 3a, Type II and V), except for Rolvedon™ (eflapegrastim‐xnst). This is reasonable as the half‐lives of linear PEGs with size in this range are short as shown in Figure 3c (data are based on a murine study after intravenous administration).^38^ BLAs are the major type of such type of PEGylated therapeutics (Type V), whereas three NDAs are approved for PEGylated NPs (Type II). Large PEG molecules with molecular weights over 10 kDa (Type III and IV) are the most popular PEGs used in clinically approved therapeutics leading to 24 approved products. These two squares (Type III and IV) also have the most diverse types of PEGylation, including 90% (19 out of 21) of approved mono‐PEGylated products (red circles in Figure 3a), all three PEGylated multimer (blue dots in Figure 3a) and all eight branched PEG‐conjugated drugs (shaded area in Figure 3a). PEGs from this range are advantageous for mono‐PEGylation given its long circulation time as shown in Figure 3c.

### 3.3 Approved indications for PEGylated drugs

**FIGURE 4:**
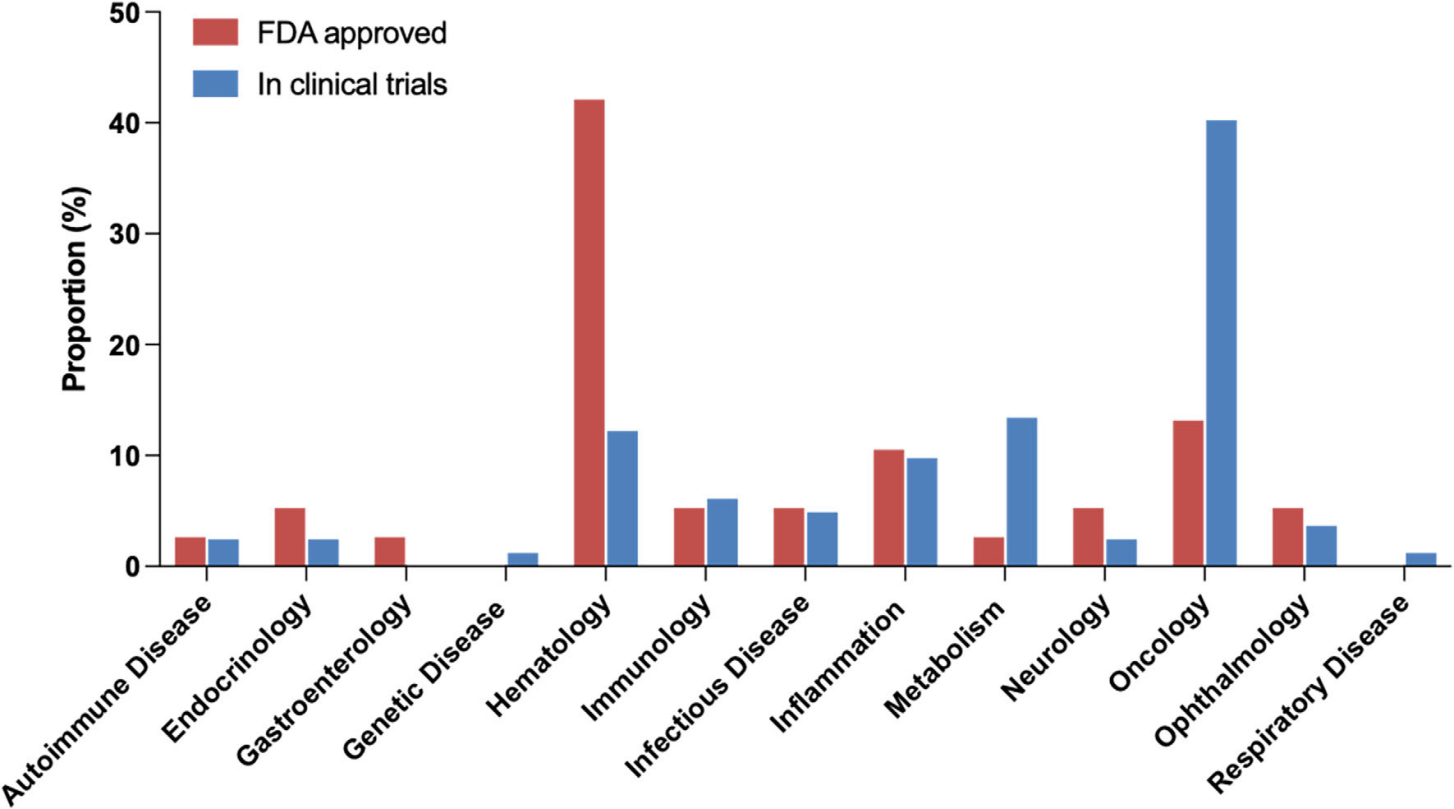
Therapeutic areas of FDA approved PEGylated therapeutics and those in active clinical trials.

Hematological diseases dominate the approved indications of PEGylated drugs (Table 1, Figure 4). A total of four coagulation factor‐based PEGylated drugs (i.e., Adynovate®, Jivi™, Rebinyn®, Esperoct®) have been approved so far for the treatment of bleeding disorders: hemophilia A or B, after intravenous administration. For other hematological disorders, one drug (Besremi™) was approved for conditions involving elevated blood cell counts (i.e., polycythemia vera), and 11 products are developed to treat disorders with decreased numbers of cells in the blood, including anemia (low levels of red blood cells),^39^ paroxysmal nocturnal hemoglobinuria (hemolytic anemia and other manifestations),^40^, ^41^ and neutropenia (low levels of neutrophils).^42^ For the indication related to neutropenia, eight PEGylated G‐CSF were approved to help with infections due to febrile neutropenia in patients who are receiving myelosuppressive chemotherapy. Based on the clinical studies, Neulasta® (pegfilgrastim, PEGylated G‐CSF) functions as a neutrophil growth factor, can restore neutrophil levels after a once‐per‐chemotherapy cycle dosing (s.c.), while a daily injection of non‐PEGylated filgrastim is needed.^22^, ^23^ The two drugs approved for anemia associated with chronic kidney disease, Omontys™ (peginesatide) and Mircera™ (methoxy polyethylene glycol‐epoetin beta), are not recommended to treat anemia associated with chemotherapy because of the increased mortality in related clinical trials. Omontys™ (peginesatide) was voluntarily recalled by the manufacturers in 2013, because of serious acute hypersensitivity reactions and associated fatalities (0.02%).^43^, ^44^ According to research performed later, such hypersensitivity reactions are likely caused by subvisible nanoparticles associated with phenol used in the commercialized multi‐use vial formulation, rather than the drug substance itself.^45^, ^46^, ^47^

PEGylated drugs have been used to treat different types of cancers. For instance, Sylatron™ (peginterferon alfa‐2b) was approved as an adjuvant therapy for melanoma after the primary treatment with surgery.^48^ Doxil® (doxorubicin HCl liposome) has multiple indications including ovarian cancer, acquired immunodeficiency syndrome (AIDS)‐related Kaposi's sarcoma and multiple myeloma (in combination with bortezomib). Onivyde™ (irinotecan liposome) in combination with 5‐fluorouracil and leucovorin is indicated to treat a type of pancreatic cancer, named metastatic adenocarcinoma of the pancreas. It should be noted that the indication for liposomal irinotecan (Onivyde™) is not approved for non‐liposomal irinotecan (Camptosar). Two PEGylated L‐asparaginase are developed for the treatment of acute lymphoblastic leukemia through depleting plasma asparagine and thus selectively killing leukemic cells.

PEGylation has also been used to develop therapeutics for other diseases with diverse indications, routes of administration and modes of action as shown in Table 1 and Figure 4. Notable examples include hepatitis B (Pegasys™), hepatitis C (Pegasys™ and Pegintron™), rheumatoid arthritis (Cimzia™), gout (Krystexxa®), multiple sclerosis (Plegridy™), and adenosine deaminase‐deficient severe combined immunodeficiency (Adagen™ and Revcovi™). Among them, peginterferon alfa‐2a (Pegasys™), peginterferon alfa‐2b (Pegintron™, for hepatitis C) and Certolizumab pegol (Cimzia™) are on the World Health Organization's List of Essential Medicines. With direct antiviral agents entering the clinic, peginterferon alfa‐2b (Pegintron™) is not a recommended treatment option for patients with hepatitis C.^49^, ^50^ PEGylated therapeutics are also developed as orphan drugs to treat rare diseases, such as Palynziq™ (pegvaliase‐pqpz) for phenylketonuria and Elfabrio® (pegunigalsidase alfa‐iwx) for Fabry disease. Palynziq™ is also considered as the first‐in‐class medication approved by the FDA.^51^ The recent approval of Syfovre™ (pegcetacoplan) represents the first FDA‐approved treatment for geographic atrophy secondary to age‐related macular degeneration, a leading cause of blindness.^52^, ^53^

### 3.4 Investigational indications for approved PEGylated drugs

Given the commercial success of several approved PEGylated therapeutics, a considerable number of clinical trials have explored the use of a currently approved therapeutic for an alternative indication. Since approved therapeutics have already demonstrated their safety and efficacy in humans through clinical trials, the investigation for their approval for an additional indication follows a more straightforward path than a new therapeutic. In addition, if commercialized, they will likely meet good manufacturing practice (GMP) standards. We have reviewed the current clinical trial landscape for previously approved PEGylated therapeutics in Table 2.

**TABLE 2:** Investigational indications of approved drugs.

Trade name [generic name]	Approved indications	Investigated indications	ClinicalTrials.gov identifier (phase)	Sponsor	Status (year)
Cimzia™ [Certolizumab pegol]	Crohn's Disease, Rheumatoid arthritis, Psoriatic arthritis, Ankylosing spondylitis	Chronic plaque psoriasis	NCT04123795 [3]	UCB, Inc.*	Recruiting (2019)
		Antiphospholipid Syndrome in Pregnancy, Lupus Anticoagulant Disorder	NCT03152058 [2]	David Ware Branch	Recruting (2017)
		Axial Spondyloarthritis	NCT02552212 [3]	UCB, Inc.*	Completed (2020)
		Interstitial Cystitis	NCT02497976 [3]	ICStudy, LLC	*Completed (2018)*
		Juvenile Idiopathic Arthritis	NCT01550003 [3]	UCB, Inc.*	Active, not recruiting (2012)
Krystexxa® [Pegloticase]	Chronic gout	Tumor Lysis Syndrome	NCT04745910 [4]	M.D. Anderson Cancer Center	Recruting (2021)
		Type 2 Diabetes	NCT03899883 [2]	University of Colorado, Denver	Recruiting (2019)
Onivyde™ [Irinotecan liposome]	Metastatic adenocarcinoma of the pancreas post gemcitabine treatment	Recurrent Solid Tumors	NCT04901702 [2]	St. Jude Children's Research Hospital*	Recruiting (2021)
		Small Cell Lung Cancer	NCT03088813 [3]	Ipsen*	Active, not recruiting (2017)
		Small Cell Lung Cancer	NCT05049863 [2]	Washington University School of Medicine	Recruiting (2021)
		Refractory Solid Tumors	NCT03810742 [1]	PharmaEngine	Active, not recruiting (2019)
		Gastrointestinal Cancer	NCT05277766 [1]	University Hospital, Ghent	Recruting (2022)
		Esophageal Squamous Cell Carcinoma	NCT03719924 [2]	Federation Francophone de Cancerologie Digestive	Recruiting (2018)
		Neuroendocrine Carcinoma	NCT03837977 [2]	The Christie NHS Foundation Trust	Active, not recruiting (2019)
		Neuroendocrine Carcinoma	NCT03736720 [2]	Roswell Park Cancer Institute	*Active, not recruiting (2018)*
		High Grade Glioma	NCT02022644 [1]	University of California, San Francisco	*Active, not recruiting (2013)*
		Biliary Tract Cancer	NCT03785873 [1]	University of Michigan Rogel Cancer Center	*Active, not recruiting (2018)*
		Pancreatic, colorectal, gastroesophageal, or biliary cancer that has spread to other places in the body (metastatic)	NCT03337087 [2]	Academic and Community Cancer Research United	Active, not recruiting (2017)
		Gastroenteropancreatic neuroendocrine tumors	NCT05385861 [2]	National Health Research Institutes, Taiwan	Not yet recruting (2022)
		Malignant Solid Neoplasm	NCT02631733 [1]	National Cancer Institute (NCI)	Active, not recruiting (2015)
		Childhood CNS Tumor	NCT03554707 [Early Phase 1]	SynerGene Therapeutics, Inc.	Not yet recruting (2018)
Besremi™ [Ropeginterferon alfa‐2b‐njft]	Polycythemia vera	Primary and Secondary Myelofibrosis	NCT02370329 [2]	Mayo Clinic	Active, not recruiting (2015)
		Pre‐fibrotic myelofibrosis	NCT05731245 [2]	National Taiwan University Hospital	Recruiting (2023)
		Hepatitis D	NCT05467553 [2]	National Taiwan University Hospital	*Not yet recruting (2022)*
		Essential Thrombocythemia	NCT05482971 [2]	PharmaEssentia Corp*	Recruiting (2022)
Oncaspar™ [Pegaspargase]	Acute lymphoblastic leukemia	NK/T‐cell Lymphoma of Nasal Cavity	NCT04414969 [2]	Hunan Cancer Hospital	Recruiting (2020)
		Extranodal NK‐T‐Cell Lymphoma	NCT02085655 [3]	Huiqiang Huang	Unknown (2014)
Doxil® [Doxorubicin HCl liposome]	Ovarian cancer, Multiple myeloma, AIDS‐related Kaposi's Sarcoma	Breast cancer	NCT05748834 [2]	SCRI Development Innovations, LLC	Not yet recruiting, (2023)
		Relapsed Sarcomas	NCT05210374 [1]	Case Comprehensive Cancer Center	Recruiting (2022)
		Secondary central nervous system lymphoma	NCT03964090 [2]	National Cancer Institute (NCI)	Recruiting (2019)
		Primary Central Nervous System Lymphoma	NCT02203526 [1]	National Cancer Institute (NCI)	Recruiting (2014)
Fulphila™ [Pegfilgrastim‐jmdb]	Infection during chemotherapy	Aplastic Anemia treatment	NCT01624805 [2]	M.D. Anderson Cancer Center	Recruting (2012)
Macugen™ [Pegaptanib sodium]	Neovascular (wet) age‐related macular degeneration	Diabetic Macular Oedema	NCT01175070 [4]	University of Oxford	*Completed (2010)*
		Diabetic Macular Edema	NCT01486238 [4]	Valley Retina Institute	Completed (2011)
		Proliferative Diabetic Retinopathy, Diabetic Macular Edema	NCT00446381 [Unknown]	Lawson Health Research Institute	*Completed (2007)*
		Proliferative Diabetic Retinopathy	NCT01486771 [4]	Valley Retina Institute	*Unknown (2011)*
		Proliferative diabetic retinopathy	NCT01487070 [1]	Retina Institute of Hawaii	Completed (2011)
Plegridy™ [Peginterferon beta‐1a]	Multiple sclerosis	COVID‐19 infection	NCT04552379 [3]	Pontificia Universidad Catolica de Chile	Completed (2020)
Pegasys™ [Peginterferon alfa‐2a]	Chronic Hepatitis C (CHC), chronic hepatitis B, Cirrhosis and compensated liver disease, CHC/HIV coinfection	Chronic Myelogenous Leukemia	NCT02736721 [3]	Roche*	Completed (2016)
		Myeloproliferative Disorders	NCT00452023 [2]	M.D. Anderson Cancer Center	Active, not recruiting (2007)
		Colon Cancer	NCT04798612 [2]	Zealand University Hospital	Not yet recruiting (2021)
		Cutaneous Squamous Cell Carcinoma	NCT05729139 [1]	Baptist Health South Florida	Not yet recruiting (2023)
		Chronic Hepatitis B	NCT04225715 [2]	Roche*	Recruiting (2020)
		Chronic Hepatitis B	NCT05244057 [2]	Shanghai HEP Pharmaceutical Co., Ltd.	Recruiting (2022)
Neulasta® [Pegfilgrastim]	Infection during chemotherapy	Type 1 Diabetes Mellitus	NCT02505893 [2]	Ospedale San Raffaele	Active, not recruiting (2015)
		Aplastic Anemia	NCT01624805 [2]	M.D. Anderson Cancer Center	Recruiting (2012)
Asparlas™ [Calaspargase pegol‐mknl]	Acute lymphoblastic leukemia	Pancreatic Adenocarcinoma	NCT05034627 [1]	OHSU Knight Cancer Institute	Recruiting (2021)

* Company sponsoring the clinical trial is the same company holding the FDA approval for the original approved indication of the drug (as seen in Table 1).

The largest number of ongoing clinical trials are for the drug Onivyde™, originally approved for the treatment of metastatic adenocarcinoma, marketed by Ipsen. These ongoing trials aim to either seek approval for an additional cancer type or for a combination therapy involving other anti‐cancer agents. Ipsen is involved as a sponsor or collaborator in clinical trials that are now actively exploring the role of Onivyde™ for indications including small cell lung cancer, neuroendocrine carcinoma, high grade glioma, and biliary tract cancer. A few other indications for which Onivyde™ is being investigated by other companies include gastrointestinal cancer and childhood CNS tumor. Doxil®, approved by the FDA in 1995 for ovarian cancer, multiple myeloma and AIDS related Kaposi's sarcoma, still persists in clinical trials for approvals for breast cancer, relapsed sarcomas and central nervous system lymphomas. Other anti‐cancer PEGylated therapeutics investigated for additional indications include Oncaspar™ and Asparlas™, both originally approved for acute lymphoblastic leukemia and now being investigated for NK/T‐cell lymphoma of nasal cavity and pancreatic adenocarcinoma respectively.

In addition to cancer, three hematological drugs Besremi™ (original indication: polycythemia vera), Fulphila™ and Neulasta® (original indications: acute lymphoblastic leukemia) are in active clinical trials for new indications including hepatitis D, aplastic anemia and type I diabetes respectively. Krystexxa®, originally approved in 2010 for the treatment of gout, is now being investigated for tumor lysis syndrome and type II diabetes. The use of Cimzia™, originally approved in 2010 for inflammatory disorders is being extended to high‐risk pregnancy as well as additional inflammatory disorders like chronic plaque psoriasis and juvenile idiopathic arthritis. Lastly, Roche's Pegasys™, originally approved in 2002 for chronic hepatitis C warrants special attention as it is now being investigated for a set of diverse indications that comprise myeloproliferative disorders, cutaneous squamous cell carcinoma, and colon cancer.

## 4 PEGYLATED AGENTS UNDER CURRENT CLINICAL TRIALS

**FIGURE 5:**
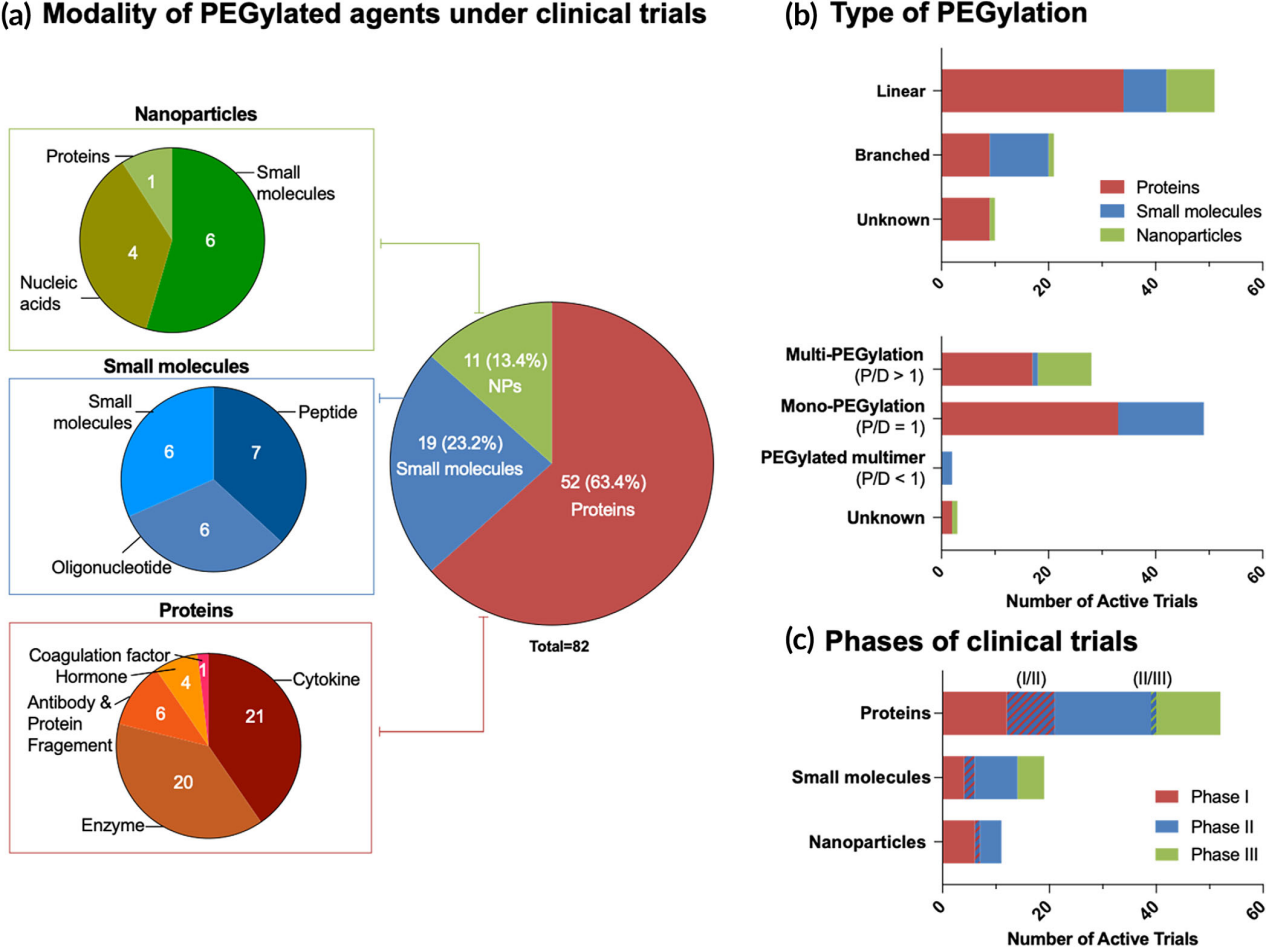
PEGylated therapeutics under active clinical trials. (a) The modality of investigational PEGylated drugs. (b) Types of PEGylation used in FDA‐approved drugs. (c) Phases of clinical trials. NPs, nanoparticles; P/D, the molar ratio of PEG to drug; PEG, polyethylene glycol.

The success of PEGylated therapeutics in the clinic continues to encourage and inspire new PEGylated products. In this subsection, we provide a snapshot of the landscape of current clinical trials of new investigational PEGylated agents as of May 2023 by searching on clinicaltrials.gov using keywords of “PEG OR PEGylated OR Pegol OR Polyethylene Glycol” and status of “recruiting, not yet recruiting, active, not recruiting, or enrolling by invitation studies.” The returned entries (over 800) were further screened manually to only include those evaluating investigational PEGylated agents. A total of 82 active clinical trials were identified and listed in Table 3. Figure 5 also summarizes the overall analysis of these PEGylated agents.

**TABLE 3:** PEGylated agents under active clinical trials.

Code name [generic name]	Parent drug [drug size]	PEG topology [PEG size]	P/D	Investigated indications	ClinicalTrials.gov identifier [phase]
Proteins					
BMS‐931699 [Lulizumab pegol]	Anti‐CD28 domain antagonist antibody (12 kDa)	Branched (40 kDa)	1	Living‐Donor Kidney Transplant, Kidney Transplant Recipients	NCT04066114 [Ph 1/2]
STK‐012	Alpha/beta‐biased engineered IL‐2 (15.4 kDa)	Branched (2 arms) (40 kDa)	1	Advanced Solid Tumor	NCT05098132 [Ph 1]
NKTR‐214 [Bempegaldesleukin]	IL‐2 (15.4 kDa)	Branched (2 arms) (20 kDa)	6	Melanoma	NCT03635983 [Ph 3]
PEG‐EPO	Erythropoietin (18.4 kDa)	Linear (30 kDa)	1	Renal Anemia	NCT05629598 [Ph 2]
PEG‐rhG‐CSF [Pegfilgrastim]	G‐CSF (19 kDa)	Linear (20 kDa)	1	Breast Cancer	NCT04477616 [Ph 2]
				Pediatric Cancer	NCT04547829 [Ph 2]
HHPG‐19K [Mecapegfilgrastim]	G‐CSF (19 kDa)	Linear (19 kDa)	1	Chemotherapy‐induced Neutropenia, Lymphoma	NCT04460508 [Ph 2]
Xinruibai [Pegfilgrastim]	G‐CSF (19 kDa)	Not found (19 kDa)	1	Nasopharyngeal Carcinoma	NCT05222009 [Ph 1]
P1101 [Ropeginterferon alfa‐2b]	IFN α‐2b (19.3 kDa)	Branched (2 arms) (40 kDa)	1	Polycythemia Vera	NCT04655092 [Ph 3]
PEG IFN α‐2b	IFN α‐2b (19.3 kDa)	Linear (12 kDa)	1	Cirrhosis of Liver Due to Hepatitis B	NCT03969017 [Ph 2]
Lambda [Peginterferon Lambda‐1A]	IFN λ (19.6 kDa)	Linear (20 kDa)	1	COVID‐19	NCT04967430 [Ph 3]
					NCT04354259 [Ph 2]
					NCT04534673 [Ph 2]
				Hepatitis D	NCT05070364 [Ph 3]
					NCT02765802 [Ph 3]
AOP2014 [Ropeginterferon alfa‐2b]	Proline‐IFN α‐2b (20 kDa)	Branched (2 arms) (40 kDa)	1	Polycythemia Vera	NCT03003325 [Ph 2]
DZP [Dapirolizumab pegol]	Anti‐CD40L Fab' antibody fragment (20 kDa)	Linear (20 kDa)	1	Systemic Lupus Erythematosus	NCT04976322 [Ph 3]
					NCT04294667 [Ph 3]
PEG‐ENDO [PEGylated endostatin]	Endostatin (20 kDa)	Not found (20 or 40 kDa)	1	Solid Tumors	NCT04413227 [Ph 1]
[Y‐shape pegylated somatropin]	Somatropin (22 kDa)	Branched (2 arms) (40 kDa)	1	Growth Hormone Deficiency	NCT04513171 [Ph 2/3]
[PEG‐somatropin]	Somatropin (22 kDa)	Branched (2 arms) (40 kDa)	1	Dwarfism	NCT03255694 [Ph 2]
ADI‐PEG 20 [Pegargiminase]	Arginine deiminase (46 kDa)	Linear (20 kDa)	Multiple	Acute Myeloid Leukemia	NCT05001828 [Ph 1]
				Soft Tissue Sarcoma	NCT05813327 [Ph 1/2]
				Non‐Small Cell Lung Cancer Small‐cell Lung Cancer	NCT05616624 [Ph 1/2]
				Soft Tissue Sarcoma	NCT05712694 [Ph 3]
				Glioblastoma Multiforme	NCT04587830 [Ph 1]
				Nonalcoholic Steatohepatitis	NCT05842512 [Ph 2]
				Hepatocellular carcinoma	NCT05317819 [Ph 3]
				Obesity	NCT05829239 [Ph 2]
PEGPH20 [Pegvorhyaluronidase alfa]	Hyaluronidase (60 kDa)	Linear (30 kDa)	1	Metastatic Pancreatic Adenocarcinoma	NCT01959139 [Ph 1/2]
TVT‐058, OT‐58 [Pegtibatinase]	hCBS enzyme (63 kDa)	Linear (20 kDa)	Multiple	Homocystinuria	NCT03406611 [Ph 2]
SSS11 [PEGylated urate oxidase]	Urate oxidase (137 kDa)	Linear (20 kDa)	1	Gout	NCT04047394 [Ph 1]
SEL‐037 [Pegsiticase]	Urate oxidase (137 kDa)	Not found (20 kDa)	Multiple	Chronic Gout	NCT04513366 [Ph 3]
					NCT04596540 [Ph 3]
[PEGylated Urate Oxidase]	Urate Oxidase (Not found)	Not found	Not found	Hyperuricemia	NCT05226013 [Ph 1]
FR104 [PEGylated Anti‐CD28 Fab’]	Anti‐CD28 Fab’ (Not found)	Branched (2 arms) (40 kDa)	1	Kidney Transplantation	NCT04837092 [Ph 1/2]
				Healthy Volunteers	NCT05238493 [Ph 1]
AEB1102 [Pegzilarginase]	Cobalt‐substituted human Arginase (110 kDa (est.))	Linear (5 kDa)	36 (est.)	Arginase I Deficiency, Hyperargininemia	NCT03921541 [Ph 3]
					NCT03378531 [Ph 2]
					NCT05676853 [Ph 3]
PegC [Pegcrisantaspase]	Erwinase (140 kDa (tetramer))	Not found (5 kDa)	Multiple	Leukemia	NCT04526795 [Ph 1]
PegC [Pegcrisantaspase]	Erwinase (141 kDa (tetramer))	Not found (5 kDa)	Multiple	Relapsed or Refractory Acute Myeloid Leukemia	NCT04526795 [Ph 1]
THOR‐707, SAR444245 [PEGylated recombinant non‐alpha IL‐2]	Non‐alpha IL‐2 (15.5 kDa (est.))	Linear (30 kDa)	1	Squamous Cell Carcinoma of Head and Neck	NCT05061420 [Ph 2]
				Classic Hodgkin Lymphoma, Diffuse Large B‐cell Lymphoma	NCT05179603 [Ph 2]
				Malignant Melanoma, Squamous Cell Carcinoma of Skin	NCT04913220 [Ph 1/2]
				Pleural Mesothelioma, Non‐small Cell Lung Cancer	NCT04914897 [Ph 2]
				Advanced and metastatic gastrointestinal cancer	NCT05104567 [Ph 2]
				Metastasis	NCT04009681 [Ph 1/2]
				Plasma Cell Myeloma Refractory	NCT04643002 [Ph 1/2]
ACN00177 [Pegtarviliase]	Cystathionineγ‐lyase (178 kDa (tetramer))	Linear (5 kDa)	Unknow	Homocystinuria Due to Cystathionine Beta‐Synthase Deficiency	NCT05154890 [Ph 1/2]
B1344 [PEGylated rh‐mutated fibroblast growth factor 21]	rh‐mutated fibroblast growth factor 21 (22 kDa est.)	Not found (20 kDa)	1	Nonalcoholic Steatohepatitis	NCT05655221 [Ph 1]
FRSW117	Coagulation Factor VIII‐Fc fusion protein (220 kDa est.)	Not found	Not found	Severe Hemophilia A	NCT05265286 [Ph 2]
Small molecules					
DFP‐14927	Radgocitabine (0.252 kDa)	Branched (4 arms) (40 kDa)	0.25	Solid Tumors	NCT03943004 [Ph 1]
PLX038 [PEGylated SN38]	SN38 (0.392 kDa)	Linear (40 kDa)	1	Small Cell Lung Cancer, Extra‐Pulmonary Small Cell Carcinomas	NCT04209595 [Ph 1/2]
				Platinum‐resistant Ovarian, Primary Peritoneal, and Fallopian Tube Cancer	NCT05465941 [Ph 2]
JK‐1201I [PEG‐Irinotecan]	Irinotecan (0.587 kDa)	Linear (20 kDa)	0.3333	Small Cell Lung Cancer (SCLC)	NCT05158491 [Ph 2]
AZD0466	AZD4320 (0.946 kDa)	Dendritic (32 arms) (2.1 kDa (per arm))	Multiple (1 drug per arm)	Non‐Hodgkin Lymphoma	NCT05205161 [Ph 1/2]
NLY01 [PEGylated exenatide]	Exenatide (4.2 kDa)	Branched (Trimeric) (50 kDa)	1	Parkinson Disease	NCT04154072 [Ph 2]
				Type 2 Diabetes	NCT04159766 [Ph 2]
PB‐119 [PEGylated exenatide]	Exenatide (4.2 kDa)	Linear (23 kDa)	1	Type 2 Diabetes	NCT04504396 [Ph 3]
					NCT04504370 [Ph 3]
					NCT05328843 [Ph 1]
BAY1097761 [PEG‐ADM Inhale]	Adrenomedullin (6 kDa)	Linear (40 kDa)	1	Acute Respiratory Distress Syndrome	NCT04417036 [Ph 2]
AON‐D21	L‐configured aptamer (12.8 kDa)	Branched (2‐arms) (40 kDa)	1	Healthy	NCT05018403 [Ph 1]
					NCT05343819 [Ph 1]
EPO‐018B [Pegol‐Sihematide]	Erythropoietin‐derived Peptide (2.5 kDa est.)	Branched (2 arms) (40 kDa)	0.5	Non‐Dialysis‐Dependent Chronic Kidney Disease	NCT03903809 [Ph 3]
HurlutinTM Lu‐177 [Lu‐177‐DOTAGA‐PEG‐IAC]	Lu‐177‐DOTAGA (0.652 kDa)	Linear (linker) (Not found)	1	Breast Cancer Stage IV	NCT04469127 [Ph 2]
Zimura [Avacincaptad pegol]	Anti‐C5 RNA aptamer (13 kDa)	Branched (2 arms) (43 kDa)	1	Stargardt Disease 1	NCT03364153 [Ph 2]
				Geographic Atrophy, Macular Degeneration	NCT04435366 [Ph 3]
					NCT05536297 [Ph 3]
NOX‐A12 [Olaptesed pegol]	L‐stereoisomer RNA oligonucleotide (15 kDa)	Branched (2 arms) (40 kDa)	1	Metastatic Pancreatic Cancer, solid tumors	NCT04901741 [Ph 2]
Nanoparticles					
HF1K16 [PEGylated liposome of ATRA]	All‐Trans Retinoic Acid (0.3 kDa)	Linear (2 kDa)	Multiple	Solid Tumor	NCT05388487 [Ph 1]
Promitil [PEGylated liposome of mitomycin‐C Lipid‐based prodrug]	Mitomycin‐C (0.334 kDa)	Linear (2 kDa)	Multiple	Cancer, Gastro‐Intestinal Intraepithelial Neoplasia	NCT04729205 [Ph 1]
Talidox, TLD‐1 [PEGylated Liposome of doxorubicin]	Doxorubicin (0.543 kDa)	Linear (2 kDa)	Multiple	Advanced Solid Tumors	NCT03387917 [Ph 1]
ThermoDox® [Lyso‐thermosensitive Liposomal Doxorubicin]	Doxorubicin (0.543 kDa)	Linear (2 kDa)	Multiple	Relapsed/refractory solid tumors (children and young adults)	NCT02536183 [Ph 1]
				Relapsed/refractory solid tumors	NCT04791228 [Ph 2]
H1ssF_3928 [VRC‐FLUNPF099‐00‐VP]	H1ssF_3928 mRNA Vaccine (size not found)	Linear (2 kDa)	Multiple	Influenza	NCT05755620 [Ph 1]
NTLA‐2001	CRISPR/Cas9 gene editing system (size not found)	Linear (2 kDa)	Multiple	Hereditary transthyretin amyloidosis with polyneuropathy (ATTRv‐PN) and cardiomyopathy (ATTRv‐CM) or wild type cardiomyopathy (ATTRwt‐CM)	NCT04601051 [Ph 1]
NTLA‐2002	CRISPR/Cas9 gene editing system (size not found)	Linear (2 kDa)	Multiple	Hereditary Angioedema	NCT05120830 [Ph 1/2]
FVIII‐PEGLip [PEGLip with Simoctocog alfa]	FVIII Simoctocog alfa (170 kDa est.)	Linear (2 kDa)	Multiple	Hemophilia A with Inhibitor	NCT04592692 [Ph 2]
Nano‐QUT [Quercetin‐encapsulated PLGA‐PEG nanoparticles]	Quercetin (0.3 kDa)	Not found	–	Oral Cancer	NCT05456022 [Ph 2]
GEN‐1	IL‐12 Plasmid (size not found)	Branched (on PEI) (0.55 kDa per arm)	Multiple	Ovarian Cancer	NCT03393884 [Ph 2]

### 4.1 Modalities of investigational PEGylated products

Similar to the trend seen in approved products, the dominant modality of PEGylated therapeutics being investigated in active clinical trials constitutes proteins (Figure 5a). There are currently 52 active trials evaluating PEGylated proteins, accounting for 63% of the total identified trials. Cytokines and enzymes constitute the bulk of these investigational PEGylated proteins, accounting for 40% (21) and 38% (20) of the trials respectively. Given the success of approved PEGylated G‐CSF products, a variation of newer PEGylated G‐CSF product is being tested in clinical trials. For instance, while all the FDA‐approved products use the amine aldehyde conjugation to link G‐CSF to PEG, the product HHPG‐19 K utilizes the thiol maleimide chemistry to link the N‐terminus of the G‐CSF to PEG. New cytokines being investigated as therapeutics following PEGylation include IFN λ and recombinant interleukins. IFN λ following covalent attachment to a 20 kDa linear PEG molecule is being investigated for COVID‐19 and Hepatitis D in five different clinical trials. While PEGylated forms of IFN α‐2a, IFN α‐2b and IFN β‐1a have been approved by the FDA, PEGylated IFN λ is not currently approved or authorized by the FDA for use. Recombinant non‐alpha interleukins (IL‐2 to IL‐8) have been attached to a linear 30 kDa PEG via strain‐promoted azide—alkyne click chemistry and are currently being investigated in seven independent clinical trials for different types of cancers. The PEGylated interleukin, THOR‐707, contains an unnatural amino acid to which the non‐cleavable PEG group is attached. This generates a homogenous, safer and more potent IL‐2 that can be administered intravenously.^54^, ^55^ Following cytokines, PEGylated enzymes are the most prominent proteins being investigated. There are eight ongoing clinical trials for the novel anti‐cancer enzyme, Pegargiminase, which comprises arginine‐deiminase (ADI) conjugated with multiple PEG (linear, 20 kDa). The efficacy of this PEGylated enzyme is determined by its ability to deplete circulating arginine levels.^56^ Additional novel PEGylated enzymes in clinical trials and their investigated indications include hyaluronidase (cancer), human cystathionine β‐synthase (homocystinuria), urate oxidase (hyperuricemia), cobalt‐substituted arginase (Hyperargininemia), Erwinia asparaginase (leukemia) and cystathionine γ‐lyase (homocystinuria). Other PEGylated protein modalities that are in clinical investigation include hormones and antibody fragments. One PEGylated coagulation factor (FVIII) and one PEGylated monoclonal antibody (lulizumab) are also being investigated as an anti‐hemophilic agent and a Treg (regulatory T cell) modulator, respectively.

Small molecular PEGylated therapeutics account for 23% of the total investigational PEGylated drugs. These drugs are balanced between PEGylated small molecules (six clinical trials), oligonucleotides (six clinical trials) and peptides (seven clinical trials). The PEGylated small molecules are all anti‐cancer agents (radgocitabine, SN38, irinotecan, AZD4320 and Lu‐177‐DOTAGA‐PEG‐IAC) which are PEGylated to improve the half‐life of the therapeutic. An L‐configured aptamer and an anti‐C5 RNA aptamer both of which prevent complement activation, and an RNA oligonucleotide for the treatment of metastatic pancreatic cancer are the PEGylated oligonucleotides in clinical trials. Among peptides, exenatide is actively evaluated in five different clinical trials that involve the linkage of the peptide with two different PEG topologies (50 kDa trimeric branched and 23 kDa linear). These PEGylated exenatide molecules are in clinical trials for the treatment of Parkinson's disease and Type 2 diabetes. An erythropoietin derived peptide and adrenomedullin are two other PEGylated peptides in clinical trials that have not been explored previously.

Lastly, NPs contribute to 13% of the PEGylated drugs' clinical trials, with one coagulation factor, six small molecules and four nucleic acids under active investigation. A PEGylated liposome encapsulating coagulation factor VIII is in Phase 2 trials as an anti‐hemorrhagic agent. PEGylated NPs with active small molecules and their investigated indications include PEGylated liposome of all‐trans retinoic acid (solid tumor), PEGylated liposome of mitomycin‐C (cancer), PEGylated liposome of doxorubicin (solid tumor) and quercetin‐encapsulated PLGA‐PEG NPs (oral cancer). Following the success of mRNA vaccines encapsulated in PEGylated NPs for COVID‐19, an mRNA vaccine for influenza with a similar PEGylated NP structure is being investigated in a Phase I study. Two CRISPR/Cas9 gene editing systems and an IL‐12 plasmid are the other nucleic acids that are part of investigational PEGylated NP therapeutics.

### 4.2 Type of PEGylations

Various types of PEGylation strategies have been utilized to synthesize these investigational PEGylated products, including similar strategies used in the FDA‐approved products and some novel PEG structures. For multi‐PEGylation (including PEGylated NPs), the majority of them use linear PEGs with three exceptions (i.e., NKTR‐214, AZD0466, and GEN‐1). Among them, AZD0466 is a novel dendrimer‐based molecule construct containing multiple linear PEGs and AZD4302, a small molecular drug, covalently attached to each arm of the dendrimer.^57^ Aided by the monodispersed dendrimer, this therapeutic agent features a defined polymer/drug ratio, specific drug conjugation sites and identical sizes, significantly minimizing the pharmacologic and structural heterogeneity issues faced by multi‐PEGylation.

Mono‐PEGylated agents are the major type of products being evaluated clinically, accounting for 60% of the active trials (Table 3 and Figure 5b). Among them, 29 trials involve linear PEG‐based agents and 17 trials contain products conjugated with branched PEGs. This category includes some novel PEG structures that have not been used in FDA‐approved products. NLY01 (PEGylated exenatide), for instance, contains a trimeric PEG (3 arm branched) with molecular weight of 50 kDa which is conjugated to exenatide, a GLP‐1r agonist.^58^ Conjugating trimeric PEG to the C‐terminal of exenatide is reported to be beneficial in prolonging the circulation half‐life, enhancing the receptor binding affinity and increasing proteolytic stability, compared to native or other PEGylated exenatide.^59^, ^60^

In addition to the aforementioned AZD0466, there are several PEGylated agents containing multiple therapeutic moieties. EPO‐018B, for instance, consists of two peptide moieties conjugated to one end of the 2‐arm branched PEG. DFP‐14927 contains a 4‐arm PEG with the therapeutic entity DFP‐10917 (an analog of the nucleoside deoxycytidine) attached at each end, leading to a PEG/drug ratio of 0.25.^61^ JK‐1201I, comprises three equivalents of irinotecan attached to a linear 20 kDa PEG via a biodegradable oligo‐peptidyl linker.^62^ Unlike the short PEG used in the approved product Movantik™, a 20 kDa PEG is used to improve the pharmacokinetics and solubility of irinotecan. But because of the long PEG chains used, a cleavable linkage between drug and polymer is used to liberate the free drugs, enabling them to function at the target cells. The trivalent conjugation design can improve the load of active drugs in the final conjugate, another common issue faced by the PEGylation of small molecule drugs.

Figure 3b summarizes the sizes of PEG used for conjugation and the corresponding active pharmaceutical ingredient. No agents using short PEGs (up to 1 kDa) were found under active clinical trials, and only 16 active trials evaluate PEGylated products using PEG with moderate sizes (1–10 kDa). Of these, the majority are 2 kDa linear PEGs to modify liposome or LNPs, containing either small molecular drugs or biomacromolecules. The rest are 5 kDa PEG‐based PEGylated proteins, as used in the first FDA‐approved product Adagen™. PEG molecules with molecular weights over 10 kDa continue their ascendance, accounting for 80% of the active trials listed in Table 3 and Figure 3b. Compared to the FDA approved products (Figure 3a), an increased number of PEGylated small molecules in Type III are being evaluated in the clinical trials (Figure 3b), indicating the strong interests in using PEG to modify and deliver small molecular agents. For Type IV PEGylated proteins, only 20 kDa PEGs are used for multi‐PEGylation, but PEGs with varied size (ranging from 12 to 43 kDa) have been utilized to develop mono‐PEGylated proteins. The Type IV square also sees most Phase 3 clinical trials (11 trials) compared to other types of PEGylation, potentially indicating a higher number of PEGylated proteins entering the market in the near future.

### 4.3 Investigational indications

Hematology and oncology are the two main therapeutic areas of ongoing clinical trials, similar to that of FDA approved products. Metabolism is the third area that has a significant proportion of active clinical trials, a marked increase from its share in FDA approved PEGylated products (Figure 4). Oncology dominates the therapeutic area by accounting for 41% of the total clinical trials of PEGylated therapeutics. Of these, the PEGylated non‐alpha interleukin‐2 (IL‐2) named THOR‐707 are roughly 15.5 kDa interleukins conjugated to a single, linear 30 kDa PEG molecule. These are being tested for different cancers in independent clinical trials, including classic Hodgkin lymphoma, malignant melanoma, esophageal squamous cell carcinoma, gastric cancer, colorectal cancer, plasma cell myeloma and pleural mesothelioma. ADI‐PEG‐20 is an arginine deiminase molecule (46 kDa) attached to 13–21 linear 20 kDa PEG molecules that is also being tested in independent clinical trials for different types of cancers, including acute myeloid leukemia, glioblastoma, hepatocellular carcinoma, lung carcinoma, soft tissue sarcoma and uveal melanoma. In addition, ThermoDox, a thermosensitive formulation of doxorubicin in a PEGylated liposome, is being investigated for soft tissue sarcoma in Phases 1 and 2.

For metabolic disorders, six investigational PEGylated products are studied in active clinical trials to address enzyme deficiencies in certain diseases, including hyperargininemia (arginase deficiency), homocystinuria (cystathione beta synthase deficiency), hyperuricemia (urate oxidase deficiency). Due to the role of ADI‐PEG‐20 in catabolizing arginine and improving insulin sensitivity, it is being tested as a treatment for obesity. Lastly, a PEGylated form of the anti‐diabetic peptide exenatide is in four independent clinical trials for the treatment of Type 2 diabetes.

Hematological indications make up roughly 12% of the total investigated indications for PEGylated agents in active clinical trials. PEGylated G‐CSF is active in four different clinical trials to treat neutropenia associated with chemotherapy. This is likely due to the commercial success of FDA approved PEGylated G‐CSF. The two antihemophilic agents in clinical trials include FVIII in a PEGylated liposome and a PEG recombinant FVIII‐Fc fusion protein. To treat polycythemia vera, two variations of PEGylated IFN α‐2b are under investigation. Finally, for the treatment of anemia, a PEGylated EPO and a PEGylated EPO derived peptide are under clinical investigation. Thus, all trials in the therapeutic area of hematology recapitulate indications that already have an FDA approved product, namely neutropenia, hemophilia, polycythemia vera and anemia.

In addition to these major therapeutic areas, other diverse indications fall in the realm of inflammation (gout and hepatitis), immunology (prevention of transplant rejection and complement inhibition), and infectious disease (COVID‐19 and influenza). The detailed breakdown of investigational PEGylated therapeutics based on therapeutic indication is shown in Table 3 and Figure 4.

### 4.4 Stages of clinical trials

Current clinical trials of investigational PEGylated agents span from Phase I to Phase III, as shown in Figure 5c and Table 3. This subsection aims to highlight novel PEGylated agents that just entered clinical trials, as well as those showing promising results in the later stage of clinical trials.

A total of 26 PEGylated products are under Phase I clinical trials (in 35 trials, including 13 trials indicated for both Phases I and Phase II). Among them, a novel PEGylated fibroblast growth factor 21 analog (B1344) is developed to treat nonalcoholic steatohepatitis, a common disease without approved medications.^63^, ^64^ Other notable products in this stage include a PEGylated small molecule (i.e., DFP‐14927) for patients with solid tumors, a novel liposomal formulation of doxorubicin (i.e., TLD‐1), and two CRISPR/Cas9 gene editing systems (i.e., NTLA‐2001 and NTLA‐2002).

Of the 43 Phase II ongoing clinical trials (including those with additional phases), about 65% are assessing PEGylated proteins. THOR‐707, for instance, is a novel PEGylated investigational IL‐2 in seven active clinical trials (Table 3).^65^ From their interim clinical data, THOR‐707 is tolerated by cancer patients and showed antitumor efficacy, especially in increasing CD8 T and NK cells treated with or without pembrolizumab and cetuximab.^66^ There are eight PEGylated small molecules and five PEGylated NPs that have entered Phase II clinical trials, including the two camptothecin derivative‐based antitumor drugs (i.e., PLX038 and JK‐1201I), and a PLGA‐PEG NP‐based formulation with quercetin encapsulated.

While no PEGylated NP‐based therapeutic agents are found under active Phase III clinical trials, 3 PEGylated small molecules (5 trials) and 8 PEGylated proteins (14 trials) have moved forward to Phase III trials (Table 3). Among them, Zimura is a novel mono‐PEGylated anti‐C5 RNA aptamer, developed as a complement C5 inhibitor for treating geographic atrophy secondary to age‐related macular degeneration.^67^ While no results have been posted yet, the FDA has granted Breakthrough Therapy and Priority Review status for Zimura, underscoring its high promises in treating these patients over the current therapies.^68^, ^69^ Another notable product is AEB1102 (Pegzilarginase). It is a multi‐PEGylated cobalt‐substituted human enzyme arginase I,^70^ developed for patients with arginase I deficiency.^71^ AEB1102 has received multiple FDA designations such as Fast Track, Breakthrough Therapy, Orphan Drug, and Rare Pediatric Disease.^72^ While the primary endpoint of the current Phase III trial has been met based on a report from the sponsor, Aeglea BioTherapeutics,^73^ in 2022, FDA issued a Refusal to File letter for pegzilarginase, requesting additional information on its effectiveness.^74^ Other intriguing agents in this stage include PEGylated arginine deiminase (pegargiminase) for soft tissue sarcoma^75^ and hepatocellular carcinoma,^76^ PEGylated IFN λ (peginterferon lambda‐1A) for hepatitis D^77^, ^78^ and COVID‐19,^79^ and PEGylated anti‐CD40L Fab' antibody fragment (Dapirolizumab pegol) for systemic lupus erythematosus.^80^

## 5 SELECT FAILURES

Despite the notable achievements of PEGylated agents in the pharmaceutical industry, it is important to acknowledge that many investigational PEGylated agents failed in getting regulatory approval. This subsection aims to analyze some representative PEGylated agents that have been discontinued either because they cannot improve the effectiveness over the control group, or because of potential side effects, as summarized in Table 4.

**TABLE 4:** Selected failures of PEGylated agents.

Code name [generic name]	Parent drug [drug size]	PEG topology [PEG size]	P/D	Intended indications	ClinicalTrials.gov identifier [phase, first‐posted year]	Status and reasons for discontinuation
Proteins						
NKTR‐358 [Rezpegaldesleukin]	IL‐2 [15.4 kDa]	Unknown	Multiple	Systemic lupus erythematosus	NCT04433585 [Ph 2, 2020]	Did not meet its primary endpoint; potential further development for other conditions.^137^
AM0010 [Pegilodecakin]	IL‐10 [36 kDa]	Unknown [5‐30 kDa]	1	Pancreatic cancer (incombination with FOLFOX)	NCT02923921 [Ph 3, 2016]	Did not meet its primary endpoint of overall survival; potential further development for other conditions.^138^
ARX201	rh‐growth hormone [22 kDa]	Unknown [30 kDa]	1	Growth Hormone Deficiency	NCT00778518 [Ph 2, 2008]	Drug development stopped due to PEG accumulation in ependymal cells of choroid plexus.^139^
PHA‐794428	rh‐growth hormone [22 kDa]	Branched [40 kDa]	1	Growth Hormone Deficiency	NCT00308464 [Ph 2, 2006], NCT00314938 [Ph 2, 2006]	Drug development stopped, due to cases of injection‐site lipoatrophy.^139^
Small molecules						
NKTR‐181 [Oxycodegol]	Oxycodone [0.315 kDa]	Linear [0.28 kDa]	1	Pain	NCT02367820 [Ph 3, 2015], NCT02362672 [Ph 3, 2015]	Demonstrated efficacy and safety, but failed to get FDA approval due to concerns about the its potential for misuse or abuse, potential effects when snorted or injected, and potential for liver toxicity; further development is stopped.^90^
NKTR‐102 [Etirinotecan Pegol]	SN‐38 [0.392 kDa]	Branched (4 arms) [20 kDa]	0.25	Breast cancer with Brain Metastases	NCT01492101 [Ph 3, 2011], NCT02915744 [Ph 3, 2016]	Did not meet its primary endpoint of overall survival; further development is ongoing.^140^
EZ‐246 [Pegamotecan]	Camptothecin [0.348 kDa]	Linear [40 kDa]	0.5	Cancer of Stomach, Gastroesophageal Cancer, Soft Tissue sarcoma.	NCT00079950 [Ph 2, 2004], NCT00080002 [Ph 2, 2004]	Did not provide safety benefits over irinotecan; further development is stopped.^141^
Nanoparticles						
MM‐302 [Antibody‐modified, PEGylated Liposomal Doxorubicin]	Doxorubicin [0.544 kDa]	Linear [2 kDa]	Multiple	HER2 Positive Breast Cancer	NCT02213744 [Ph 2/3, 2014]	Cannot meet its primary endpoint of progression‐free survival.^92^
LiPlaCis [Liposomal cisplatin]	Cisplatin [0.301 kDa]	Linear [2 kDa]	Multiple	Metastatic Breast Cancer, Prostate Cancer and Skin Cancer	NCT01861496 [Ph 1/2, 2013]	Did not provide safety benefit over standard cisplatin formulations; reformulation is required for further development.^142^

Although using PEGylation to extend therapeutic circulation time has been particularly fruitful for biopharmaceuticals, a number of PEGylated proteins failed to show superiority during clinical studies. For instance, developing a long‐acting human growth hormone for the treatment of growth hormone deficiency has been explored for many years, leading to one product, Skytrofa™ (Lonapegsomatropin‐tcgd), receiving FDA approval in 2021. However, at least three PEGylated human growth hormones have been discontinued during the clinical studies. The clinical trial for ARX201, developed by attaching a 30‐kDa PEG to growth hormone, was stopped, since studies showed the accumulation of PEG in choroid plexus epithelial cells,^81^ which has been widely discussed in terms of the safety of PEGylated products (as mentioned below).^37^ Similarly, the study of PHA‐794428, a recombinant growth hormone conjugated with a 40 kDa branched PEG, was terminated due to the occurrence of injection‐site lipoatrophy, which was presumably due to the lipolytic effects of growth hormone.^82^, ^83^ In the case of FDA‐approved product, Skytrofa™, a 4‐arm branched PEG was attached to the human growth hormone via a cleavable TransCon linker that can release human growth hormone after injection and no difference was found in terms of injection site reactions, compared to unmodified growth hormone,^84^ implying that a releasable PEGylation design is indeed beneficial for this type of therapeutic protein. While PEGylation of interleukins showed favorable pharmacokinetics,^85^, ^86^ two PEGylated interleukins: PEGylated IL‐2 (Rezpegaldesleukin) and PEGylated IL‐10 (Pegilodecakin), were unsuccessful in meeting their primary endpoints for treating systemic lupus erythematosus^85^ and pancreatic cancer,^87^ respectively.

The clinical translation of PEGylated small molecules, in particular chemotherapeutics, represents one challenging task in the field of PEGylation. Pegamotecan, for example, is one of the early developments of PEGylated chemotherapies to improve solubility, safety, and potency of camptothecin. Its Phase 2 clinical trial was terminated due to its similar toxicity profile compared to irinotecan.^88^ From the PEGylation point of view, pegamotecan consists of two equivalents of camptothecin attached to the two ends of a 40 kDa PEG. Although camptothecin can be released via hydrolysis,^89^ the large PEG size used contributes to 98% of the molecular mass of the final conjugate. Thus, a significant amount of conjugates are needed to be administered to achieve the intended drug dosages. While using short or multi‐arm PEG can address this limitation, etirinotecan pegol, a product using a 4‐arm 20 kDa PEG with one drug molecule attached to each arm, failed to show efficacy in proving overall survival in treating breast cancer with brain metastases. Another recently discontinued PEGylated small molecule is oxycodegol which contains a short, specific PEG chain attached to oxycodone. Although Phase I‐III studies demonstrated its safety and therapeutic potentials in treating pain, it failed to get FDA approval due to concerns about its potential for misuse or abuse, potential effects when snorted or injected, and risk for liver toxicity.^90^

In terms of PEGylated NPs, LiPlaCis is a PEGylated liposomal cisplatin, which failed to improve the safety of standard cisplatin formulations based on Phase I/II studies.^91^ MM‐302, a novel HER2‐targeted, PEGylated liposomal doxorubicin with anti‐HER2 antibody attached on the liposomal surface, was developed to treat HER2 Positive Breast Cancer, but failed to meet its primary endpoint of progression‐free survival.^92^, ^93^

## 6 CHALLENGES

While PEGylation is being widely used and explored for drug modifications, there are some potential challenges in clinically translating PEGylated products, which need to be considered in order to further advance the field of PEGylated therapeutics. In this subsection, we highlight the issues associated with the use of PEG molecules (e.g., safety and immunogenicity), the scientific and technological obstacles of PEGylation, and the challenges associated with regulatory approvals.

### 6.1 Safety and immunogenicity

Although PEG was historically considered to be a biologically inert material, recent studies have shown that approximately 20%–70% of humans with no known PEG exposure have anti‐PEG antibodies that can recognize the polymer.^94^, ^95^, ^96^, ^97^ In addition, owing to the daily exposure to products containing PEG such as cosmetics, pre‐existing anti‐PEG antibodies are also present in many healthy humans.^97^, ^98^ As a result, reports have indicated loss of therapeutic efficacy due to the immune response elicited by the PEGylated therapeutics^99^, ^100^ by causing accelerated blood clearance and a reduction in half‐life.^101^, ^102^, ^103^, ^104^ Additionally, hypersensitivity reactions, which are a set of undesirable reactions caused by the immune system to PEGylated drugs have been reported, further drawing attention to the risks associated with the PEG component of PEGylated therapeutics.^105^ Such infusion reactions or CARPA (Complement activation‐related pseudoallergy) have been seen after the administration of Jivi™, where patients developed anti‐PEG antibodies and hypersensitivity reactions like urticaria, angioedema and dyspnea.^106^ It is worth noting that these adverse reactions are seen not only in PEGylated proteins, but also in PEGylated NPs. For instance, 45% of cancer patients who received Doxil® developed hypersensitivity reactions that included shortness of breath, flushing, and dizziness.^107^, ^108^ For the recently approved Pfizer/BioNTech BNT162b2 and Moderna mRNA‐1273 vaccines, several cases with PEG‐related allergic reactions have been reported, with and without anaphylaxis, the symptoms of which include hives, diarrhea, dizziness, shortness of breath and irregular heart rate.^106^, ^109^ The factors influencing adverse reactions to PEG such as the molecular weight of PEG, morphological properties, surface charge and route of administration have been summarized in recent reports.^106^, ^110^, ^111^, ^112^ Finally, PEG associated cytoplasmic vacuolation has also been observed after the administration of PEGylated drugs, such as Cimzia™ and Somavert™, giving rise to accumulation of PEG in cells.^113^, ^114^ Although severe side effects of such vacuolation have not been reported, the consequence of long‐term accumulation in cases of life‐long therapies remains to be seen.

As a result of growing concerns about PEG's immunogenicity, there have been efforts in search of alternatives to PEG in order to address some of these deficiencies. Some of the commonly explored alternatives include known biocompatible polymers like polyvinylpyrrolidone (PVP) and poly(2‐oxazoline). For instance, PVP is an attractive alternative to PEG owing to properties like high hydration, good biocompatibility and low immunogenicity. However, its use is limited by its non‐biodegradability and poorly understood immunological properties.^115^ Polyoxazolines, which are thermosensitive polymers having good solubility in both organic and aqueous solvents have been grafted to several NPs to provide stealth properties. However, the difficulty and high cost of synthesis acts as a major barrier to its use as a PEG alternative.^116^, ^117^ Other explored alternatives to PEG include hydrophilic polymers like polyglycerols, poly(N‐(2‐Hydroxypropyl) methacrylamide) (PHPMA), natural polymers like glycosaminoglycans (GAGs) and zwitterionic materials like poly(carboxybetaine). However, their usage as a PEG alternative has been restricted by several limitations which have been reviewed elsewhere.^112^, ^115^ In addition to using polymers, PASylation (Pro, Ala, Ser) and XTENylation (Ala, Asp, Gly, Pro, Ser, Thr) are strategies in which peptide chains are used as linkers or half‐life enhancing agents.^118^, ^119^ A recombinant antihemophilic product that makes use of XTEN called ALTUVIIIO has been recently approved by the FDA.^120^ However, despite comparable physicochemical and pharmacological properties to PEG, most of these PEG alternatives have demonstrated varied immunological properties, indicating that the alternatives are not immunologically inert.^112^

### 6.2 Scientific and technological challenges

PEGylation as a technology has significantly evolved over the past three decades, overcoming numerous technological obstacles along the way. The field has progressed from the random, multi‐PEGylation using short and linear PEG molecules, as used in the first PEGylated protein (i.e., Adagen™), to site‐specific PEGylation using diverse types of PEGs (e.g., sizes and topologies). Nevertheless, there are still several scientific and technological challenges that require careful consideration. One of them is the polydisperse nature of PEG molecules. Similar to many other polymers, commercial PEGs are generally polydisperse homologous mixtures spanning a wide range of molecular weights. This heterogeneity in molecular size of the PEG is passed on to the PEGylated therapeutics and could be further amplified if multiple and random amounts of PEGs are conjugated to each therapeutic entity. Such a polydisperse mix of PEGylated therapeutics then leads to batch‐to‐batch variations in crucial properties including solubility, clearance rate, binding affinities, posing challenges for manufacturing and quality control and thus creating new hurdles for regulatory approvals. A solution to this challenge would be to use monodisperse PEGs, which are only available commercially for low molecular weight PEGs (e.g., <1 kDa). Developing monodisperse PEGs with higher molecular weights that have generally been sought for PEGylation is one urgent task for generating homogenous, monodisperse PEGylated therapeutics, a topic that has been enthusiastically pursued in the PEGylation field in recent years.

The covalent conjugation of PEGs to therapeutic agents leads to another inherent challenge, which is the potential detrimental effect on their activity and therapeutic potency. The access of the substrate to the active site of the drug can be hampered by the presence of PEG. The affinity of the drug to its target site may also be reduced due to changes in conformation and surface electrostatic charge. Studies have shown that based on the PEGylation method and molecular weight of the PEG, the activity retained by the PEGylated product can vary between 7% and 98%.^121^ Another study showed that the activity of a 19 kDa IFNα‐2a was reduced to 7% after it was conjugated to multiple 40 kDa PEGs.^122^ This was attributed to the PEGylation sites being located near active sites. This puts emphasis on the fine control of PEGylation sites and conjugation methods in general. Although site‐specific PEGylation can be achieved nowadays through using judiciously selected chemistries, or using enzymes, or introducing non‐natural amino acids with proper reaction handles into proteins,^55^, ^123^ identifying the conjugation site with minimal compensation on its activity remains the principal challenge for PEGylating proteins. Computational simulation could be an useful tool to predict the potential activity loss after site‐specific PEGylation.^124^, ^125^

The need for PEGylation of traditionally considered small molecule drugs is pressing, given that many effective small molecule drugs, such as irinotecan,^126^ suffer from undesirable pharmacokinetic profiles.^127^ However, the success rate of clinical translation of PEGylated small molecules is extremely low, thus indicating that several obstacles remain to be addressed. Although no apparent technological constraints hamper PEGylating traditional small molecule drugs, there are several drawbacks that make PEGylation a less desirable strategy for modifying small molecule drugs. First, small molecule drugs generally resist chemical modifications due to the limited functionalities and the tight structure–activity relationships. Conjugating large molecular weight PEGs to small molecule drugs leads to reduced fractions of active drugs in the final conjugates, as mentioned in the case of Pegamotecan. These issues, together with the polydispersity nature of PEGs, need to be considered when designing new PEGylated small molecules. An increase in the understanding of the structure–activity relationships of both PEGylated and native drugs in the clinic will certainly lay a strong foundation for selectively modifying these small molecules. Unlike the post‐translational modification of macromolecules, PEGylation of small molecules should be integrated during the drug synthesis given their generally low chemical modification sites. Using releasable conjugation bonds and multifunctional PEGs may also become necessary for such types of drugs, but it leads to another set of questions on how to control the timing and location of drug release without losing the benefits of PEGylation in modulating their pharmacokinetics.

### 6.3 Regulatory considerations

In addition to the aforementioned issues, there are further challenges associated with the regulatory approval process. One of them is that these chemically modified therapeutics are generally considered as new drugs or new biologics, regardless of the clinical status of the parent drugs and PEG molecules. Although a rigorous regulatory approach and comprehensive clinical evaluations are vital for ensuring patient safety, the lengthy and costly nature of this process represents a significant barrier to the development of new PEGylated therapeutics. A noteworthy advancement in this field is the FDA‐approval pathway for biosimilars, which allows manufacturers to focus on demonstrating similarity of their proposed agents to FDA‐approved reference products. This streamlined approach allows for accelerated approval without the need to establish the biosimilar's safety and effectiveness independently. A handful of PEGylated proteins have already obtained approval as biosimilars (e.g., Nyvepria™, Ziextenzo™, Udenyca™, Stimufend®, Fulphila™, and Fylnetra™), contributing to the expedited clinical translation of PEGylated biologics. Furthermore, the concept of non‐covalent PEGylation holds promise in offering similar benefits without the need for chemical modification, thus substantially reducing translational barriers associated with regulatory approval.

Additionally, the concept of non‐biological complex drugs^128^ is expected to mitigate the impact of issues related to the polydispersity of PEGs. The FDA is also currently engaged in discussions regarding the evaluation of therapeutic equivalence for complex products, which encompass drugs with complex active ingredients (e.g., polymeric compounds) that are challenging to fully characterize, identify, and quantify.^129^, ^130^, ^131^

## 7 CONCLUSION

PEGylation is a widely recognized and successful technology for extending the half‐life of therapeutic agents, having achieved remarkable clinical and commercial accomplishments. Since the FDA approval of the first PEGylated protein, Adagen™, in 1990, many PEGylated therapeutics have been approved and used in the clinic to treat a variety of diseases. To date, PEGylation technology has been applied to nearly all kinds of therapeutic agents including small molecules, nucleotides, peptides, proteins and NPs, consistently demonstrating clinical advantages in improving their pharmacokinetics and pharmacodynamics. This clinical progress of PEGylation is accompanied by significant advancements in the PEGylation design, making it possible nowadays to achieve site‐specific PEGylation using PEG molecules with desirable sizes, topologies and PEG/drug ratios. Ongoing active clinical trials testify to the continual evolution of PEGylation technology, with novel PEGylated agents continuously emerging. While challenges such as the polydisperse nature of PEG and the immunogenicity profile of PEG molecules exist, PEGylation remains the most cost‐effective and safest option for extending the half‐life of therapeutics, especially when compared to alternative polymers/technologies. This is evident in the extensive use of PEGylation in human patients over several decades and its recent successful application in large‐scale administration of PEGylated LNPs‐based COVID‐19 vaccines to the general population. These achievements underscore the favorable properties and proven track record of PEGylation as a valuable approach in therapeutic development.

Looking ahead, PEGylation will continue to have a tremendous impact on the development of new therapeutic agents, offering not only half‐life extension but also many other benefits. The field will certainly be driven by the rapid expansion of macromolecular drugs (such as proteins and peptides), as well as the emergence of LNP encapsulated mRNA therapies. PEGylation is also anticipated to play a vital role in the development of other novel therapies such as immunotherapies or combinational therapies by enabling the conjugation of different therapeutic agents onto a single PEG molecule. The progress of the field will also be facilitated by the advancements in the synthesis of monodispersed PEG with diverse architectures, as well as the commercial availability of PEG products and conjugation chemistries that are already established for clinical use. Thus, overall, the future of PEGylation holds immense promise in driving innovation and advancements in therapeutic agent development.

## AUTHOR CONTRIBUTIONS

**Yongsheng Gao:** Conceptualization (equal); data curation (equal); formal analysis (equal); writing – original draft (equal); writing – review and editing (equal). **Maithili Joshi:** Conceptualization (equal); data curation (equal); formal analysis (equal); writing – original draft (equal); writing – review and editing (equal). **Zongmin Zhao:** Conceptualization (equal); funding acquisition (equal); writing – review and editing (supporting). **Samir Mitragotri:** Conceptualization (equal); funding acquisition (equal); writing – review and editing (equal).

## CONFLICT OF INTEREST STATEMENT

Yongsheng Gao and Samir Mitragotri are inventors on patent applications related to topics discussed in the manuscript (owned and managed by Harvard University).

## Data Availability

All data are available in the main manuscript or supplementary materials. The original data of clinical trials are available upon reasonable request.
